# Rapid Characterization of Triterpene Saponins from *Zornia brasiliensis* by HPLC-ESI-MS/MS

**DOI:** 10.3390/molecules24142519

**Published:** 2019-07-10

**Authors:** Yuri Mangueira Nascimento, Lucas Silva Abreu, Ramon Leal Lima, Vicente Carlos O. Costa, José Iranildo Miranda de Melo, Raimundo Braz-Filho, Marcelo Sobral Silva, Josean Fechine Tavares

**Affiliations:** 1 Graduate Program in Natural and Synthetic Bioactive Products, Health Sciences Center, Universidade Federal da Paraíba, João Pessoa 58051-900, Paraíba, Brazil; 2 Graduate Program in Ecology and Conservation, Department of Biology, Center for Biological and Health Sciences, Campina Grande 58429-500, Paraíba, Brazil; 3 Center of Sciences and Technologies, Darcy Ribeiro Norte Fluminense State University, Campos dos Goytacazes 28013-600, Rio de Janeiro, Brazil

**Keywords:** triterpene saponins, soyasaponins, *Zornia brasiliensis*, HPLC-DAD-ESI-MS/MS

## Abstract

*Zornia brasiliensis* Vogel (Leguminosae) is a species popularly known in Brazil as “urinária”, “urinana”, and “carrapicho”, it is popularly used as a diuretic and in the treatment of venereal diseases. A specific methodology to obtain a saponin-enriched fraction and high-performance liquid chromatography coupled with diode array detection, ion trap mass spectrometry, and TOF-MS (HPLC-DAD-ESI-MS/MS) was applied for the analysis of triterpene saponins. The MS and MS/MS experiments were carried out by ionization in negative mode. Molecular mass and fragmentation data were used to support the structural characterization of the saponins. Based on retention times, high-resolution mass determination and fragmentation, 35 oleanane-triterpene saponins were tentatively identified in *Z. brasiliensis*.

## 1. Introduction

The family Leguminosae consists of approximately 770 genera and 19,500 species. It previously included only three subfamilies, but a recent study reorganized the Leguminosae into six subfamilies [1]. These include the subfamily Papilionoideae, which contains the greatest number of genera, including the genus *Zornia*.

The genus *Zornia* contains approximately 80 species that are distributed throughout the world, mainly in tropical and subtropical regions. In Brazil, the genus is represented by 36 species, 15 of which are endemic [2,3]. Studies have investigated the pharmacological activities of compounds present in species of this genus, including cytotoxic, smooth muscle-relaxing, anticonvulsant, antibacterial, antitumor, anti-inflammatory, and antioxidant activities [4,5,6,7,8,9,10,11,12]. In turn, phytochemical studies have revealed the presence of isoflavonoids [13,14].

*Zornia brasiliensis* Vogel, a species popularly known in Brazil as “urinaria”, “urinana”, and “carrapicho”, is adopted in the alternative medicine as a diuretic and in the treatment of venereal diseases [15]. *Z. brasiliensis* is distributed in the North, Northeast, Center-West and Southeast regions of Brazil. It is associated with the Amazon, Caatinga, Cerrado, and Atlantic Forest phytogeographic domains [16], but is found mainly in the Brazilian Northeast [17] and Venezuela [18]. Two pharmacological studies of this species have reported the antinociceptive activity of 7-methoxyflavone isolated from its aerial parts [19] and the antitumor activity of its essential oil [20]. A recent phytochemical study of the aerial parts of this species resulted in the identification of 14 compounds, including flavones, flavanones, isoflavonoids, pterocarpans, chalcones, and a novel glycosylated dihydrochalcone—and this chalcone showed cytotoxic activity against HL-60 leukemic cells [21].

Hyphenated techniques, such as high-performance liquid chromatography (HPLC) coupled to mass spectrometry (MS) were used for the structural analysis and characterization of saponins [22,23,24,25,26,27,28,29]. This study represents the first contribution to describe the existence of triterpene saponins from the genus *Zornia* (Leguminosae). A total of 35 saponins were detected and tentatively identified by MS and MS/MS.

## 2. Results

### 2.1. Characterization of the Compounds by HPLC-ESI-MS/MS

**Figure 1:**
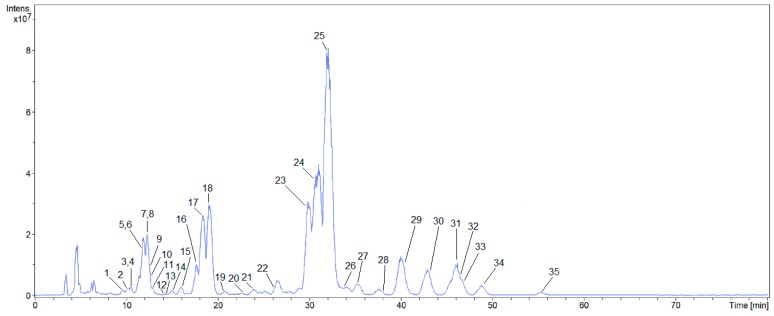
Base peak chromatogram (BPC) of the concentrated fraction of saponins obtained by ESI-MS/MS in negative mode.

Identification of triterpene saponins by high-performance liquid chromatography-electrospray ionization-tandem mass spectrometry (HPLC-ESI-MS/MS) with collision-induced dissociation (CID) under negative ESI-MS is a well-established and widely employed technique [24]. To obtain these MS/MS data, a low-resolution mass spectrometer was used; and the accurate mass was obtained by a high-resolution spectrometer, both using the same ionization parameters, and both coupled to a high-performance liquid chromatography system under the same chromatographic conditions. The 35 saponins that are present in the aerial parts of *Z. brasiliensis* and tentatively identified are shown on base peak chromatogram (Figure 1).

According to Pollier et al., after collision-induced dissociation (CID) under negative ESI-MS, the saponins undergo glycosidic cleavages, retaining the charge at the reducing end (e.g., aglycone containing fragment). In the MS/MS spectrum, daughter ions provide information on the sugar residues and the aglycone of the fragmented saponin. Sugar residues are mainly hexoses (e.g., glucose and galactose), 6-deoxyhexoses (e.g., rhamnose and fucose), pentoses (e.g., arabinose and xylose), and uronic acids (e.g., glucuronic acid and galacturonic).

The sugar residues and the aglycone could be identified from the MS/MS spectra. The typical losses represent water (−18 Da); and/or rhamnoside residue (−146 Da); and/or arabinose (−150 Da); and/or glucuronic acid (−158 Da). Therefore, the daughter aglycone ion, [Aglycone − H]^−^ is based on this type or pattern of analysis. Based on this analysis, thirty-five saponins from *Zornia braziliensis* could be tentatively identified by mass spectrometry. The MS/MS data of the tentatively identified saponins are reported in Table 1.

#### 2.1.1. Aglycones of Saponins of *Z. Brasiliensis*

**Figure 2:**
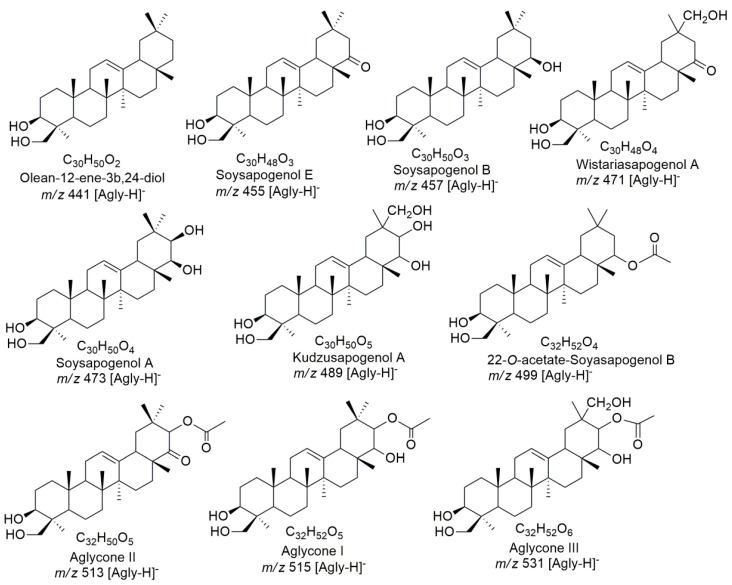
Aglycones of *Z. brasiliensis* saponins tentatively identified by mass spectrometry.

In the MS/MS fragmentation spectra obtained for the 35 saponins, ions *m/z* 441–531 Da, characteristic of triterpene aglycones, were observed. Through the relationships of the deprotonated ions of the saponins—*m/z* 631.3856–999.5135 Da, and the respective fragment ions at the *m/z* 441–531 Da interval—10 aglycones were tentatively identified (Figure 2) [24]. Although these aglycones have been detected only as daughter ions with nominal *m/z* values, their molecular formulas can be predicted by the molecular formula of the exact mass of the parent ion and by the observed losses of the sugar residues [24]. Seven of these aglycones (olean-12-ene-3β,24-diol; soyasapogenol A, B, and E; kudzusapogenol A; wistariasapogenol A; and 22-*O*-acetate-soyasapogenol B) are of the oleanane type and have been previously reported in the family Leguminosae (Figure 2) [28,30,31,32,33].

No previous report of three of these sapogenins was found in the literature. Aglycone I has a fragment ion at *m/z* 515 [Agly − H]^−^ and molecular formula C_32_H_52_O_5_, thus, we propose that aglycone would be an acetylation product of soysapogenin A (*m/z* 473), since it is possible to observe the loss of 42 Da (acetoxy) after subtraction of glycosidic residues, suggesting that acetoxy group is present in the sapogenin.

Aglycone II has a fragment ion *m/z* 513 [Agly − H]^−^ and the molecular formula C_32_H_50_O_5_. The loss of 42 Da (acetoxy), similar to that observed in 21-acetoxy-soyasapogenol A (*m/z* 515), was observed. However, aglycone II has two fewer mass units, suggesting that C-22 was oxidized to generate a ketone [34]. The ion resulting from this loss of 42 Da, *m*/*z* 471, showed distinct fragmentation of wistariasapogenol A, a difference suggested by the absence of ion *m*/*z* 439 relative to the loss of 32 Da (CH_4_O) (Figure 2). Aglycone III has an *m*/*z* 531 [Agly − H]^−^ and the molecular formula C_32_H_52_O_6_, it has been tentatively identified as an acetylated derivative of kudzusapogenol A, as evidenced by the 42 Da loss and the presence of the ion at *m*/*z* 489 (Figure 2).

This study has proposed the putative characterization of monodesmosidic saponins with the insertion of glycosides or its chains at the C-3 position, as observed in the saponin isolated and identified by NMR and supported by the literature which describes saponins present in the Leguminosae family [28,30,30,35]. The possibility of the presence of bidesmosidic saponins was removed due to the presence of fragments *m/z* 483 [M − H]^−^ referring to the union of a dHex + Hex + HexA, *m/z* 453 [M − H]^−^ dHex + Pen + HexA, *m/z* 337 [M − H]^−^ Hex + HexA, and *m/z* 307 [M − H]^−^ Pen + HexA, thus, demonstrating that the sugar chain is attached in the one position of the sapogenin, thus, excluding the possibility of bidesmosidic saponins.

#### 2.1.2. Saponins of *Z. brasiliensis*

Peaks **1** and **2** showed ions at *m/z* 925.4814 and 779.4186, respectively, thus, they are isobars of compounds **27** and **28**. However, they presented distinct fragmentation patterns that made it possible to propose that the difference between these compounds lies in their aglycones. In the tentative identification, compounds **1** and **2** have wistariasapogenol as an aglycone *m/z* 471 [Agly − H]^−^, this is proposed based on their fragmentation, since the fragment ion *m/z* 439 [Agly − H − CH_4_O]^−^ is compatible with the loss of 32 Da corresponding to the loss of methanol. This suggests oxygenation in methyl C-30 rather than C-21, a ketone in the C-22 position similar to soyasapogenol E, and one other fragment ion *m/z* 407 [Agly − H − CH_4_O − CH_4_O]^−^, which is supposedly attributed to methanol at the C-23 position. Compound **1** was putatively identified as wistariasaponin A, which was previously isolated from a species of the family Leguminosae [35]; and compound **2** was tentatively identified as Pen-HexA-wistariasapogenol A. 

Peaks **3** and **4** presented the fragment ion *m/z* 489 [Agly − H]^−^ were tentatively identified as derivatives of kudzusapogenol A, a penta-oxygenated oleanane triterpene with oxygenations at C-21 and C-22, the oxygenation at C-29 is supported by the loss of 32 Da (methanol) observed in the fragment ion *m/z* 457 [Agly − H − CH_4_O]^−^. Compound **3** was putatively identified as Pen-HexA-kudzusapogenol A and compound **4** as subproside II, a saponin isolated from *Sophorae subprostrata* (Leguminosae) [36,43].

Group A soyasaponins are bidesmosidic, with insertion of the glycosidic chains in C-3 and C-22, and can be divided into acetylated and non-acetylated [22]. They have soyasapogenol A as the aglycone, which differs structurally from soyasapogenol B in the presence of a hydroxyl group at C-21 [44]. Five saponins that possess this aglycone were putatively identified (*m/z* 473). Compound **5** was identified as soyasaponin A_3_ [37], which was described by Curl et al. as a monodesmosidic derivative of soyasapogenol A. Compound **8**, identified as Hex-HexA-soyasapogenol A, has already been described but not named [33], and compounds **6**, **7**, and **14** were putatively identified as dHex-Pen-HexA-soyasapogenol A, Pen-HexA-soyasapogenol A, and HexA-soyasapogenol A, respectively.

Peaks **9** and **10** showed the fragment ion *m/z* 531 [Agly − H]^−^, attributed to their aglycone and are here proposed as derivatives of aglycone III. It was also possible to observe the fragment ions *m/z* 489 and 457, which were attributed to the neutral loss of an acetoxy residue (42 Da) and a methanol (32 Da) of the aglycone. Peaks **9** and **10** were tentatively characterized as dHex-Pen-HexA-aglycone III and Pen-HexA-aglycone III, respectively. 

Four saponins tentatively proposed possess aglycone II (*m/z* 513). These saponins were observed in peaks **11**, **12**, **13**, and **15** and were proposed as dHex-Hex-HexA-aglycone II, Hex-HexA-aglycone II, dHex-Pen-HexA-aglycone II, and Pen-HexA-aglycone II, respectively. 

Peak **20** was tentatively identified as Hex-HexA-22-*O*-acetate-soyasapogenol B. It presented fragment ion at *m/z* 499 [Agly − H]^−^, attributed to 22-*O*-acetate-soyasapogenol B; and fragment ion *m/z* 457, attributed to the neutral loss of the acetoxy residue (42 Da) in the aglycone. 

Fragment ion at *m/z* 515 [Agly − H]^−^, attributed to this aglycone I, was observed in five compounds, **16**, **17**, **18**, **19**, and **22**, all of which had the fragment ion at *m/z* 473 [Agly − H − C_2_H_2_O]^−^, compatible with the neutral loss of 42 Da relative to the acetoxy group present in the aglycone *m/z* 515 [Agly − H]^−^. These compounds were tentatively identified as dHex-Hex-HexA-aglycone I, dHex-Pen-HexA-aglycone I, Hex-HexA-aglycone I, Pen-HexA-aglycone I, and HexA-aglycone I, respectively. 

Among soyasaponins A, B, and E, the soyasaponins of group E are the most restricted since they are considered photooxidation products of the soyasaponins of group B. The glycosidic chain at the C-3 position is the same for groups A, B, and E [41]. Peaks **21**, **31**, **32**, **33**, and **35** showed the same fragmentation pattern as that proposed for the olean-12-ene-3β,24-diol derivatives, however, the fragment ion attributed to the aglycone, *m/z* 455 [Agly − H]^−^, was proposed to be soyasapogenol E. Compound **21** was tentatively identified as soyasaponin Be [41], and **31**, **32**, and **33** were tentatively identified as soyasaponin Be', soyasaponin Bg, and soyasaponin Bg', respectively [44]. Compound **35** was tentatively characterized as hexA-soyasapogenol E. 

The soyasaponins of group B have only one glycosidic chain at the C-3 position. They are commonly divided into two groups, one group has a conjugation in C-22 with 2,3-dihydro-2,5-dihydroxy-6-methyl-4-pyrone (DDMP) and is more abundant in soybean, and the members of the other group are DDMP-nonconjugated [22]. In this study, it was possible to observe peaks **23**, **24**, **25**, **26**, and **29**, all of which had the fragment ion at *m/z* 457 [Agly – H]^−^ attributed to soyasapogenol B. Compounds **23** and **25** were tentatively identified as soyasaponins I and II, respectively [44,45,46]. Compounds **24**, **25**, and **29** could be tentatively identified as soyasaponin III (or soyasaponin Bb'), soyasaponin IV (or soyasaponin Bc'), and soyasapogenol B monoglucuronide [28,38].

**Figure 3:**
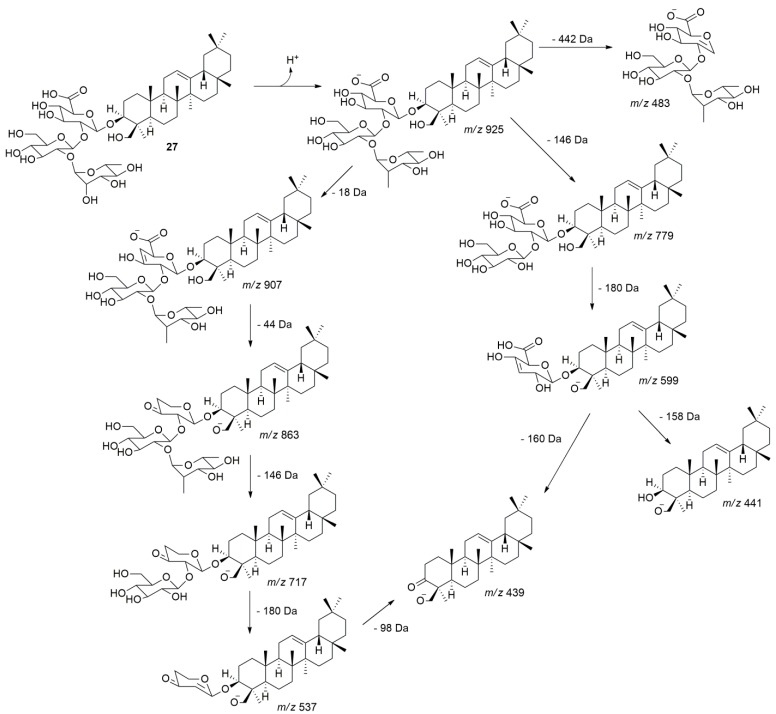
Proposed fragmentation pathway for compound **27** in negative mode ESI-MS/MS. The pathway extends similarly to that for compounds **28**, **30**, and **34**, generating the fragment ions *m/z* 441 and *m/z* 439 attributed to the aglycone olean-12-ene-3β,24-diol.

Peak **27** showed a precursor ion in *m/z* 925.5168 [M − H]^−^ and its fragmentation, the ions at *m/z* 907 [M − H − H_2_O]^−^, *m/z* 863 [M − H − H_2_O − CO_2_]^−^, *m/z* 779 [M − H − dHex]^−^, *m/z* 717 [M − H − H_2_O − CO_2_ − dHex]^−^, *m/z* 599 [M − H − dHex − Hex]^−^, *m/z* 537 [M − H − H_2_O − CO_2_ − dHex − Hex]^−^, *m/z* 483 [M − H − Agly]^−^, *m/z* 441 [M − H − dHex − Hex − HexA]^−^, and *m/z* 439 [M − H − dHex − Hex − HexA]^−^. Thus, the uronic acid residue was observed, generating a loss of 158 and/or 160 Da (Figure 3). Peaks **28**, **30**, and **34** were tentatively identified as Hex-HexA-Olean-12-ene-3β,24-diol, dHex-Pen-HexA-Olean-12-ene-3β,24-diol, and Pen-HexA-Olean-12-ene-3β,24-diol, respectively (See proposed fragmentation pathways for all compounds in the Supplementary Materials).

### 2.2. NMR Identification of Compound **25**

**Figure 4:**
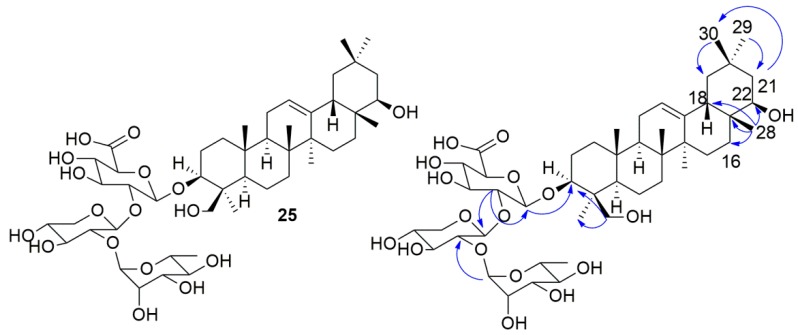
Soyasaponin II (**25**) isolated from *Z. brasiliensis*. (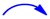) indicates some key HMBC correlations for compound **25**.

After analysis of the saponin-enriched fraction by HPLC-DAD, the major peak observed in the chromatogram was peak **25**, enabling its isolation. The compound **25** (Figure 4) has an [M − H]^−^ ion at *m/z* 911.5006 (*m/z* 911.5010, calcd.), compatible with the molecular formula C_47_H_75_O_17_. The MS^2^ experiment showed *m/z* 765 [M − H − Rha]^−^, *m/z* 615 [M − H − Rha − Ara]^−^, and *m/z* 457 [M − H − Rha − Ara − Glu]^−^ fragment ions corresponding to the consecutive losses of 146, 150, and 158 Da, respectively. In the ^13^C NMR spectrum, 7 methyls were observed attributed to the triterpenic sapogenin [δ_C_ 22.3 (Me-23), 15.3 (Me-25), 16.7 (Me-26), 25.0 (Me-27), 20.4 (Me-28), 32.7 (Me-29), 28.4 (Me-30)], a signal in δ_C_ 61.6 (C-24) characteristic of oxygenated carbon was also observed, showing that, in this structure, a Me-24 is oxygenated. Two other signals were observed in δ_C_ 121.7 (C-12) and 144.1 (C-13), attributed to the *sp2* carbons—C-12 (methine) and C-13 (nonhydrogenated), thus, this sapogenin is a triterpenic oleanane-type. The presence of a C-19 methylene carbon (δ_C_ 46.4) and C-20 (δ_C_ 30.3) suggests that this sapogenin is *β*-amyrin, which is oxygenated at the C-22 positions (δ_C_ 74.2 ) and C-3 (δ_C_ 90.0). In the ^1^H-NMR it was possible to observe the signals of 7 methyls [δ_H_ 1.08 (23-Me), 0.79 (25-Me), 0.88 (26-Me), 1.04 (Me-27), 0.74 (Me-28), 0.96 (Me-29), 0.96 (Me-30)], at the same time, a broad singlet at δ_H_ 5.16 with integral for one hydrogen, attributed to a *sp2* methyl carbon. The signals at δ_H_ 3.80 (d, *J* = 11.0 Hz, 1H) and 3.04 (m, 1H) were assigned to Ha and Hb-24, respectively, δ_H_ 3.12 (m, 1H) to H-3, and δ_H_3.23 (m, 1H) to H-22. In the ^1^H x ^13^C-HMBC spectrum, a correlation of the anomeric proton H-1′ [δ_H_ 4.13 (d, *J* = 7.5 Hz, 1H)] of *β*-glucuronic acid was observed with C-3 (δ_C_ 90.0), confirming the insertion of *β*-glucuronic acid in the C-3-position of the sapogenin. Another contour map between H-1′′′ [δ_H_ 4.97 (brs, 1H)] at three bonds distance with C-2′′ (δ_C_ 77.6) confirming the insertion of *α*-rhamnose into C-2′′ of β-arabinose. The HMBC also attributed a correlation of the proton H-2′ [δ_H_ 3.33 (m)] at two bonds distance with C-1′ (δ_C_ 103.9) and three bonds with C-1′′ (δ_C_ 100.7), thus confirming that *β*-arabinose is bonded to *β*-glucuronic acid and this insertion occurs at C-2′ glucuronic acid. Another contour map present in HMBC attributed Me-28 [δ_H_ 0.74 (s, 3H)] with C-22 (δ_C_ 74.2), C-16 (δ_C_ 28.0), C-17 (δ_C_ 37.0), and C-18 (δ_C_ 44.7) indicating that C-22 supports the hydroxyl. This information was corroborated by another contour map between H-16a [δ_H_ 1.33 (m)] and C-22 (δ_C_ 74.2). A cross-correlation was also observed between Me-29/30 [δ_H_ 0.96 (s, 6H)] with C-19 (δ_C_ 46.1), C-20 (δ_C_ 30.3), C-21 (δ_C_ 41.2), and C-29 (δ_C_ 32.7); H-19 [δ_H_ 1.67 (m) and 0.88 (m)] with C-20 (δ_C_ 30.3), C-29 (δ_C_ 32.7) and C-30 (δ_C_ 28.4) and H-21 [δ_H_ 1.30 (m)] with C-17 (δ_C_ 37.0), C-22 (δ_C_ 74.2) and C-30 (δ_C_ 28.4). It was also possible to observe, in HMBC, a three-bond correlation between H-24a [δ_H_ 3.80 (d, *J* = 11.0 Hz)], C-3 (δ_C_ 90.0) as well as Me-23 (δ_C_ 22.3). Thus, **25** corresponds to soyasaponin II (Figure 4). Comparison of the ^1^H and ^13^C-NMR spectral data of compound **25** with data from the literature corroborate this proposal. Thus, the *m/z* 457 fragment ion corresponds to the sapogenin soyasapogenol B (Figure 2) [44,45].

Considering that the isolation, purification, and elucidation of triterpene saponins are difficult and time-consuming due to the high polarity and structural similarity of these compounds [23], the present study enabled us to obtain and chemically analyze a concentrated fraction of saponins of *Z. brasiliensis* for the first time. In this study, 35 triterpene saponins were tentatively identified by HPLC-ESI-MS/MS, suggesting a homologous series. In addition, saponins have a diversification of applications such as biological and pharmaceutical, for example, immunostimulating, hypocholesterolemic, and anticarcinogenic properties, as well as applications in the food, agriculture, and cosmetics industries. This fact led to the extraction and identification of saponins in numerous species [47] due to their relevance. This is the first report of the occurrence of saponins in the genus *Zornia*, placing the species *Z. brasiliensis* in the list of the bioproducing species of this important class of secondary metabolites that are the saponins.

## 3. Materials and Methods

### 3.1. Reagents and Materials

The NMR analyses were performed on a Varian NMR system 500-MHz spectrometer (Palo Alto, CA, USA) operated at 500 MHz for ^1^H-NMR and at 125 MHz for ^13^C-NMR; and on a Bruker Ascend 400 spectrometer (Bruker, Billerica, MA, USA) operated at 400 MHz for ^1^H-NMR and at 100 MHz for ^13^C NMR. The samples were prepared in deuterated dimethyl sulfoxide (DMSO-*d*_6_) (Cambridge Isotope Laboratories, Tewksbury, MA, USA) containing TMS as internal standard. Silica gel (ART 7734, Merck, Darmstadt, Germany; particle size 0.060–0.200 mm and 70–230 mesh) was used for column chromatography (CC). To obtain the crude extract and in chromatographic fractionation, the following solvents were used: Ethanol, hexane, dichloromethane, ethyl acetate, methanol, and *n*-butanol (Tedia, Fairfield, CT, USA). For the HPLC analyses, the following solvents were used as the mobile phase: HPLC-grade acetonitrile (Tedia), ultrapure water obtained using a Milli-Q purification system (Millipore, Burlington, NJ, USA), and formic acid (Qhemis, Indaiatuba, Brazil). To obtain mass spectra by direct injection, an Ion Trap-amaZonX spectrometer (Bruker, Billerica, MA, USA) was used for electrospray ionization mass spectrometry (ESIMS, low resolution), and a micrOTOF II spectrometer was used for high-resolution ESIMS (HRESIMS, Bruker, Billerica, MA, USA) at 3.5 kV capillary voltage, ESI in negative mode, 500 V end plate offset, 8.0 psi nebulizer, dry gas (N_2_) at a flow rate of 5.0 L/h, and a temperature of 200 °C. Spectra (*m*/*z* 50–1000) were recorded every 2.0 s. Collision-induced dissociation (CID) fragmentation was achieved in auto MS/MS mode using the advanced resolution mode for MS and MS/MS mode.

### 3.2. Plant Material

The aerial parts of *Z. brasiliensis* Vogel (Leguminosae) were collected in the municipality of Serra Branca (07°29′46′′S and 36°44′36′′W; altitude, 712 m) in the state of Paraíba, Brazil, in March 2016. Collection authorization No. 53894-1 was granted by the Chico Mendes Institute for Biodiversity Conservation (ICMBio, Brasília, Brazil) through the System of Authorization and Information on Biodiversity (SISBIO, Brasília, Brazil), and access registration in the National Management System of Genetic Patrimony and Associated Traditional Knowledge (SISGEN, Curitiba, Brazil) was obtained under number ADD107E. The species was identified by botanist Dr. José Iranildo Miranda de Melo of the Paraíba State University (UEPB, Campina Grande, Brazil). An exsiccatae was deposited at Arruda Câmara Herbarium (ACAM, Campina Grande, Brazil), *Campus* I, UEPB, under the number of registration 1862.

### 3.3. Extraction and Isolation of Z. brasiliensis Constituents 

The dry and pulverized aerial parts of *Z. brasiliensis* (5 kg) were extracted with ethanol to obtain a crude ethanolic extract (CEE) which was partitioned with hexane, dichloromethane, ethyl acetate, ethyl acetate–methanol (90:10, *v*/*v*) and ethyl acetate–methanol (50:50, *v*/*v*)in order to obtain its respective fractions [21].

An aliquot (20.0 g) of the 50% ethyl acetate–methanol fraction was dissolved in 300 mL of distilled water, followed by partition with *n*-butanol (300 mL) three times. The extractive solution of the *n*-butanol phase was then concentrated on a rotary evaporator under reduced pressure at a temperature of 50 °C. The concentrated material was redissolved in methanol–ethyl acetate (1:5, *v*/*v*), and the saponins were precipitated by the addition of methanol. This material was decanted for 72 hours at room temperature. The pellet was then resuspended in methanol (200 mL) and concentrated on a rotary evaporator under reduced pressure at 40 °C. In this way, a concentrated fraction of saponins (1.1 g) was obtained [48].

### 3.4. HPLC-DAD Conditions

An analytical HPLC instrument (Shimadzu-Prominence, Kyoto, Japan) equipped with an LC-20AT pump, an SIL-20A HT auto injector, a CTO-20A oven, an SPD-M20A detector, and a CBM-20A controller unit was employed. The columns used were a Phenomenex Gemini^®^ C18 (250.0 mm × 4.6 mm internal diameter [id], filled with 5.0-µm particles, Torrance, CA, USA) and a Security Guard Gemini^®^ C18 precolumn (4.0 mm × 3.0 mm id, filled with 5.0-µm particles, Torrance, CA, USA). The elution method used water (0.1% formic acid) (A) and methanol (B). The gradient was 70.0% B (0.01 min) to 80.0% B (50 min), returning to 70.0% B (65 min), and remaining at 70.0% B until 80 min with a mobile phase flow rate of 0.6 mL/min, an injection volume of 20.0 µL, and a detection wavelength of 205 nm. The applied samples were filtered through 0.45-µm nylon membranes (Tedia, Fairfield, CT, USA).

Preparative chromatographic analyses were performed using an HPLC system (Shimadzu, Kyoto, Japan) equipped with an LC-6AD binary solvent pump, a Rheodyne injector, an SPD-M10A diode array detector, and an SCL-10A system controller. An ACE C18 column (250.0 mm × 21.2 mm id, 5.0-μm particle size) was used. The method used was similar to the method used in the analytical runs. The preparative scale injection volume was 100.0 µL, flow was conducted at 8.0 mL/min, and the detection wavelength was 205 nm. The applied samples were filtered through 0.45-µm nylon membranes (Tedia).

### 3.5. HPLC-ESI-MS/MS Conditions

A Shimadzu (Prominence, Kyoto, Japan) HPLC instrument equipped with an LC-20AT binary solvent pump, an SIL-20A auto injector, a DGU-20A degassing system, an SPD-M20A diode array detector, and a CBM-20A controller was used. A Phenomenex Gemini^®^ C18 column (250.0 mm × 4.6 mm id, filled with 5.0-µm particles) and a SecurityGuard Gemini^®^ C18 precolumn (4.0 mm × 3.0 mm id, filled with 5.0-µm particles) were used. The chromatographic method was the same as the method developed for analytical HPLC. The applied samples were filtered through 0.45-µm nylon membranes (Tedia). This HPLC instrument was coupled to an Ion Trap-amaZonX low-resolution mass spectrometer (Bruker) that used the electrospray ionization technique. The analysis parameters of the Ion-Trap spectrometer were 4.5 kV capillary, ESI in negative ion mode, a 500 V end plate offset, a 35.0 psi nebulizer, a dry gas (N_2_) flow rate of 8.0 L/h, and a temperature of 300 °C. CID fragmentation was achieved in auto MS/MS mode with the advanced resolution mode for MS and MS/MS mode. Spectra (*m*/*z* 200–1200) were recorded every 2.0 s.

For high-resolution HPLC-ESI-MS analyses, we used the HPLC instrument described above with the same column and the same chromatographic conditions. The instrument was coupled to a micrOTOF II high-resolution mass spectrometer (Bruker) that used the electrospray ionization technique. The micrOTOF II spectrometer analysis parameters were 4.0 kV capillary, ESI in negative ion mode, a 500 V end plate offset, a 35.0 psi nebulizer, a dry gas (N_2_) flow rate of 8.0 L/h, and a temperature of 300 °C. Spectra (*m*/*z* 50–1000) were recorded every 2.0 s. The sodium formate was used as the internal calibrator during the chromatographic run. The equipment resolution is ~15,000, however, when operating in HPLC-HRESIMS mode it drops to ~11,000.

## Figures and Tables

**Table 1:** Characterization of the saponins tentatively identified by HPLC-ESI-MS/MS in *Z. brasiliensis.*

Compound	T_R_ (min)	[M − H]^−^	[M − H]^−^ Calculated	Molecular Formula	Error (ppm)	Fragments (*m/z*)	Tentative Identification	Reference
**1**	10.5	925.4800	925.4802	C_47_H_73_O_18_	0.2	907, 863, 779, 717, 629, 567, 471, 453, 439, 407	Wistariasaponin A	[36]
**2**	11.1	779.4239	779.4223	C_41_H_63_O_14_	2.0	735, 629, 585, 567, 471, 439, 407	Pen-HexA-Wistariasapogenol A	-
**3**	11.2	797.4310	797.4329	C_41_H_65_O_15_	2.4	753, 647, 621, 603, 585, 489, 457, 307	Pen-HexA-Kudzusapogenol A	-
**4**		943.4912	943.4908	C_47_H_75_O_19_	−0.4	925, 881, 797, 735, 647, 585, 489, 457	Subproside II	[37]
**5**	12.5	957.5056	957.5065	C_48_H_77_O_19_	0.9	939, 895, 811, 749, 631, 569, 483, 473	Soyasaponin A_3_	[28,38]
**6**		927.4943	927.4959	C_47_H_75_O_18_	1.8	909, 865, 781, 719, 631, 569, 473, 453	dHex-Pen-HexA-Soyasapogenol A	-
**7**	13.0	781.4350	781.4380	C_41_H_65_O_14_	3.9	763, 631, 613, 569, 473, 307	Pen-HexA-Soyasapogenol A	-
**8**		811.4459	811.4485	C_42_H_67_O_15_	3.2	793, 631, 587, 473, 337	Hex-HexA-Soyasapogenol A	[39]
**9**	13.2	985.4961	985.5014	C_49_H_77_O_20_	5.3	967, 839, 821, 777, 689, 627, 531, 489, 457, 453	dHex-Pen-HexA-Aglycone III	-
**10**	13.7	839.4404	839.4435	C_43_H_67_O_16_	3.6	821, 689, 671, 627, 531, 489, 457, 307	Pen-HexA-Aglycone III	-
**11**	14.2	997.5022	997.5014	C_50_H_77_O_20_	−0.8	979, 935, 851, 789, 671, 609, 513, 483, 471	dHex-Hex-HexA-Aglycone II	-
**12**	15.1	851.4412	851.4435	C_44_H_67_O_16_	2.7	807, 671, 513, 471	Hex-HexA-Aglycone II	-
**13**	15.6	967.4901	967.4908	C_49_H_75_O_19_	0.7	949, 905, 821, 671, 653, 609, 513, 471, 453	dHex-Pen-HexA-Aglycone II	-
**14**	16.9	649.3933	649.3957	C_36_H_57_O_10_	3.8	631, 473	HexA-Soyasapogenol A	-
**15**	17.7	821.4303	821.4329	C_43_H_65_O_15_	3.2	689, 671, 653, 609, 513, 471, 307	Pen-HexA-Aglycone II	-
**16**	18.3	999.5135	999.5170	C_50_H_79_O_20_	3.6	981, 937, 853, 791, 673, 611, 515, 483, 473	dHex-Hex-HexA-Aglycone I	-
**17**	19.5	969.5074	969.5065	C_49_H_77_O_19_	−1.0	951, 907, 823, 761, 673, 611, 515, 473, 453	dHex-Pen-HexA-Aglycone I	-
**18**	19.9	853.4559	853.4591	C_44_H_69_O_17_	3.8	835, 673, 655, 611, 515, 473, 337	Hex-HexA-Aglycone I	-
**19**	20.2	823.4463	823.4485	C_43_H_67_O_15_	2.7	805, 673, 629, 611, 515, 473, 307	Pen-HexA-Aglycone I	-
**20**	23.9	837.4603	837.4642	C_44_H_69_O_15_	2.0	657,639, 595, 499, 497, 457, 455, 453	Hex-HexA-22-*O*-acetate-Soyasapogenol B	-
**21**	24.8	939.4935	939.4959	C_48_H_75_O_18_	2.6	921, 877, 793, 731, 613, 551, 483, 455	Soyasaponin Be	[22,24,25,27,29,40,41]
**22**	26.2	691.4042	691.4063	C_38_H_59_O_11_	3.1	673, 515, 473	HexA-Aglycone I	-
**23**	31.0	941.5123	941.5115	C_48_H_77_O_18_	−0.8	923, 879, 795, 733, 615, 553,	Soyasaponin I	[22,24,27,28,29,40,41]
**24**	32.6	795.4520	795.4536	C_42_H_67_O_14_	2.1	777, 733, 615, 553, 457, 337	Soyasaponin III	[27,28,29,41]
**25**	33.0	911.5006	911.5010	C_47_H_75_O_17_	0.5	893, 849, 765, 703, 615, 553,	Soyasaponin II^a^	[27,28,29,41]
**26**	34.0	765.4418	765.4431	C_41_H_65_O_13_	1.6	747, 615, 597, 553, 457, 307	Soyasaponin IV	[27,28,29,41]
**27**	36.9	925.5168	925.5166	C_48_H_77_O_17_	−0.2	907, 863, 779, 717, 599, 537, 483, 441, 439	dHex-Hex-HexA-Olean-12-ene-3β,24-diol	-
**28**	39.3	779.4562	779.4587	C_42_H_67_O_13_	3.2	599, 441, 439, 307	Hex-HexA-Olean-12-ene-3β,24-diol	-
**29**	41.3	633.3998	633.4008	C_36_H_57_O_9_	1.5	615, 457	Soyasapogenol B monoglucuronide	[28,42]
**30**	44.1	895.5043	895.5061	C_47_H_75_O_16_	1.9	877, 833, 749, 687, 599, 537, 453, 441, 439, 337	dHex-Pen-HexA-Olean-12-ene-3β,24-diol	-
**31**	47.8	793.4348	793.4380	C_42_H_65_O_14_	4.0	775, 731, 613, 551, 455, 337	Soyasaponin Be'	[24,30]
**32**	48.0	909.4843	909.4853	C_47_H_73_O_17_	1.1	891, 847, 763, 701, 613, 551, 455, 453	Soyasaponin Bg	[25,30]
**33**	48.7	763.4286	763.4274	C_41_H_63_O_13_	−1.5	719, 613, 569, 455, 307	Soyasaponin Bg'	[30]
**34**	51.1	749.4485	749.4482	C_41_H_65_O_12_	−0.5	599, 537, 441, 439, 307	Pen-HexA-Olean-12-ene-3β,24-diol	-
**35**	58.8	631.3856	631.3852	C_36_H_55_O_9_	−0.7	613, 455	HexA-Soyasapogenol E	-

Legend: HexA, uronic acid (glucuronic acid or galacturonic acid); Hex, hexose (glucose or galactose); Pen, pentose (arabinose or xylose); dHex, 6-deoxyhexose (rhamnose or fucose), ^a^ Identified by ^1^H and ^13^C-NMR. Accurate mass obtained by HPLC-HRESIMS and fragments obtained by HPLC-ESI-MS/MS.

## Supplementary Materials

Supplementary data (Figures S1–S27) associated with this article, including HRESIMS, MS/MS, 1D, and 2D-NMR spectra data and the proposed fragmentation pathways of the tentatively identified compounds can be found in the online version.
