# Supplementary material for: Rapid Characterization of Triterpene Saponins from Zornia brasiliensis by HPLC-ESI-MS/MS
Source: Molecules. 2019 Jul 10;24(14):2519. doi: 10.3390/molecules24142519 (PMC6680687; doi:10.3390/molecules24142519)
Supplement: Supplementary file 1 [file molecules-24-02519-s001.pdf]

Rapid characterization of triterpene saponins from *Zornia brasiliensis* by HPLC-ESI-MS/MS

Yuri Mangueira Nascimento [1], Lucas Silva Abreu [1], Ramon Leal Lima [1], Vicente Carlos O. Costa [1], José Iranildo Miranda de Melo [2], Raimundo Braz-Filho [3], Marcelo Sobral Silva [1], and Josean Fechine Tavares [1,*]

[1] Graduate Program in Natural and Synthetic Bioactive Products, Health Sciences Center, Universidade Federal da Paraíba, 58051-900, João Pessoa, Paraíba, Brazil; yurimangueira@ltf.ufpb.br; lucas.abreu@ltf.ufpb.br; ramonlima.fr@hotmail.com

[2] Graduate Program in Ecology and Conservation, Department of Biology, Center for Biological and Health Sciences, 58429-500, Campina Grande, Paraíba, Brazil; tournefort@gmail.com

[3] Center of Sciences and Technologies, Darcy Ribeiro Norte Fluminense State University, 28013-600 Campos dos Goytacazes, Rio de Janeiro, Rio de Janeiro, Brazil; braz@uenf.br

* Correspondence: josean@ltf.ufpb.br; Tel.: +55-83-3216-7427.

Supporting Information

	Table of contents	Page
**Table 1.**	Comparative ^{13}C and ^{1}H NMR data of **25** in DMSO-d_6 (100 and 500 MHz) of the sapogenin of Soyasaponin II	3
**Table 2.**	Comparative ^{13}C and ^{1}H NMR data of **25** in DMSO-d_6 (100 and 500 MHz) of the sugars of Soyasaponin II	4
**Figure S1.**	^{1}H-NMR (500 MHz, DMSO-d_6) spectrum of compound **25**	**5**
**Figure S2.**	^{13}C-NMR (100 MHz, DMSO-d_6) spectrum of compound **25**	**5**
**Figure S3.**	HSQC (500 x 125 MHz, DMSO-d_6) spectrum of compound **25**	**6**
**Figure S4**.	HMBC (500 x 125 MHz, DMSO-d_6) spectrum of compound **25**	**6**
**Figure S5**.	Expansion of HMBC (500 x 125 MHz, DMSO-d_6) spectrum of compound **25**	**7**
**Figure S6**.	Expansion of HMBC (500 x 125 MHz, DMSO-d_6) spectrum of compound **25**	**7**
**Figure S7**.	The HRESI-TOF-MS spectrum of compound **25**	**8**
**Figure S8**.	The ESI-MS/MS spectrum of compound **25**	**8**
**Figure S9**.	The ESI-MS/MS spectrum of compounds **1** and **2**	**9**
**Figure S10**.	The ESI-MS/MS spectrum of compounds **3** and **4**	**10**
**Figure S11**.	The ESI-MS/MS spectrum of compounds **5**, **7**, **8** and **14**	**11**
**Figure S12**.	The ESI-MS/MS spectrum of compounds **9** and **10**	**12**

**Figure S13**.	The ESI-MS/MS spectrum of compounds **11**, **12**, **13** and **15**	**13**
**Figure S14**.	The ESI-MS/MS spectrum of compounds **16**, **18**, **19** and **22**	**14**
**Figure S15**.	The ESI-MS/MS spectrum of compound **20**	**15**
**Figure S16**.	The ESI-MS/MS spectrum of compounds **21**, **31**, **32**, **33** and **35**	**16**
**Figure S17**.	The ESI-MS/MS spectrum of compounds **24**, **26**, and **29**	**17**
**Figure S18**.	The ESI-MS/MS spectrum of compounds **27**, **28**, **30** and **34**	**18**
**Figure S19.**	Fragmentation pathway of compounds **28** (*m/z* 779.4562), **30** (*m/z* 895.5043) and **34** (*m/z* 749.4485) having olean-12-ene-3β,diol (*m/z* 441 or 439) as aglycone in negative mode ESI-MS/MS	**19**
**Figure S20.**	Fragmentation pathway of compounds **21** (*m/z* 939.4935), **31** (*m/z* 793.4348), **32** (*m/z* 909.4843), **33** (*m/z* 763.4286) and **35** (*m/z* 631.3856), which have soyasapogenol E (*m/z* 455) as aglycone, in negative mode ESI-MS/MS	**19**
**Figure S21**	Fragmentation pathway of compounds **23** (*m/z* 941.5123), **24** (*m/z* 795.4520), **25** (*m/z* 911.5006), **26** (*m/z* 765.4418) and **29** (*m/z* 633.3998), which possess soyasapogenol B (*m/z* 457) as aglycone, in negative mode ESI-MS/MS	**20**
**Figure S22.**	Fragmentation pathway of compounds **5** (*m/z* 957.5056), **8** (*m/z* 811.4459), **6** (*m/z* 927.4943), **7** (*m/z* 781.4350) and **14** (*m/z* 649.3933), which have soyasapogenol A (*m/z* 473) as aglycone, in negative mode ESI-MS/MS	**20**
**Figure S23**	Fragmentation pathway of compounds **16** (*m/z* 999.5135), **18** (*m/z* 853.4559), **17** (*m/z* 969.5074), **19** (*m/z* 823.4463) and **22** (*m/z* 691.4042), which have 21-acetoxy-soyasapogenol A (*m/z* 515) as aglycone, in negative mode ESI-MS/MS	**21**
**Figure S24.**	Fragmentation pathway of compounds **11** (*m/z* 997.5022), **12** (*m/z* 851.4412), **13** (*m/z* 967.4901) and **15** (*m/z* 821.4303), which have aglycone I (*m/z* 513) as aglycone, in negative mode ESI-MS/MS	**21**
**Figure S25.**	Fragmentation pathway of compounds **1** (*m/z* 925.4800) and **2** (*m/z* 779.4239), which have wistariasapogenol A (*m/z* 471) as aglycone, in negative mode ESI-MS/MS	**22**
**Figure S26.**	Fragmentation pathway of compounds **3** (*m/z* 797.4310) and **4** (*m/z* 943.4912), which have kudzusapogenol A (*m/z* 485) as aglycone, in negative mode ESI-MS/MS	**22**
**Figure S27.**	Fragmentation pathway of compounds **9** (*m/z* 985.4961) and **10** (*m/z* 839.4404) having aglycone II (*m/z* 531) and that of compound **20** (*m/z* 837.4603), which has 22-*O*-acetate-soyasapogenol B (*m/z* 499) as aglycone, in negative mode ESI-MS/MS	**23**

Table 1. Date of ^{13}C and ^{1}H NMR data of **25** in DMSO-d_6 (100 and 500 MHz) of the sapogenin of Soyasaponin II [30].

	HSQC		HMBC	
**C**	δ_C	δ_H	$^2J_{CH}$	$^3J_{CH}$
4	43.3	-	-	-
8	41.8	-	-	-
10	36.0	-	-	-
13	144.3	-	-	-
14	43.3	-	-	-
17	37.0	-	-	-
20	30.3	-	-	-
**CH**				
3	90.0	3.12 (m)	-	-
5	55.5	nd	-	-
9	46.9	1.47 (m)	C-8/C-10/ C-11	C-1/Me-25/ Me-26
12	121.7	5.16 (sl)	-	C-9/C-18
18	44.7	nd	-	-
22	74.2	3.23 (sl)	-	-
**CH_2**				
1	38.4	1.52 (m); 0.89 (m)	C-10	C-9
2	25.4	1.65 (d, *J* = 9.5 Hz); 0.92 (m)	-	-
6	18.1	1.52 (m); 1.30 (m)	C-7	C-10
7	32.7	1.44 (m); 1.28 (m)	C-8	Me-26
11	23.3	1.28 (m)	-	-
15	25.6	1.99 (m); 0.91 (m)	-	-
16	28.0	1.33 (m); 0.95 (m)	C-17	C-22
19	46.1	1.67 (m); 0.88 (m)	C-18	Me29/ Me-30
21	41.2	1.30 (m)	C-20/C-22	C-17/C-30
24	61.6	3.80 (d, *J* = 11 Hz); 3.04 (m)	-	C-3/C-23
**CH_3**				
23	22.3	1.08 (s)	C-4	C-3/C-5/ C-24
25	15.3	0.79 (s)	C-10	C-1/C-5
26	16.7	0.88 (s)	C-8	C-7
27	25.0	1.04 (s)	-	C-8
28	20.4	0.74 (s)	C-17	C16/C18/C-22
29	32.7	0.96 (s)	C-20	C-19/C-21
30	28.4	0.96 (s)	C-20	C-19/C-21

Table 2. Date of ^{13}C and ^{1}H NMR data of **25** in DMSO-d_6 (100 and 500 MHz) of the sugars of Soyasaponin II [30].

	HSQC		HMBC	
**C**	δ_C	δ_H	$^{2}J_{CH}$	$^{3}J_{CH}$
1’	103.9	4.13 (d, J = 6.0 Hz)	-	C-3
2’	77.2	3.33 (m)	C-1’	C-1”
3’	77.1	3.30 (m)	-	-
4’	73.8	3.17 (m)	-	-
5’	76.4	3.12 (m)	-	-
6’	172.8	-		-
1’’	100.7	4.68 (d, J = 7.0 Hz)	-	-
2’’	77.6	3.16 (m)	-	C-1”’
3’’	70.7	3.49 (m)	-	-
4’’	69.5	3.23 (m)	-	
5’’	65.4	3.60 (dd, J = 4.5 and 11.0 Hz) e 2.92 (t, J = 11.0 Hz)	-	-
1’’’	100.3	4.97 (sl)	C-2”’	C-2”’/C-5”’
2’’’	70.7	3.65 (sl)	-	-
3’’’	72.5	3.05 (m)	-	-
4’’’	72.6	3.13 (m)	-	-
5’’’	68.1	3.51(m)	-	-
6’’’	17.9	1.06 (d)	C-5”’	C-4”’

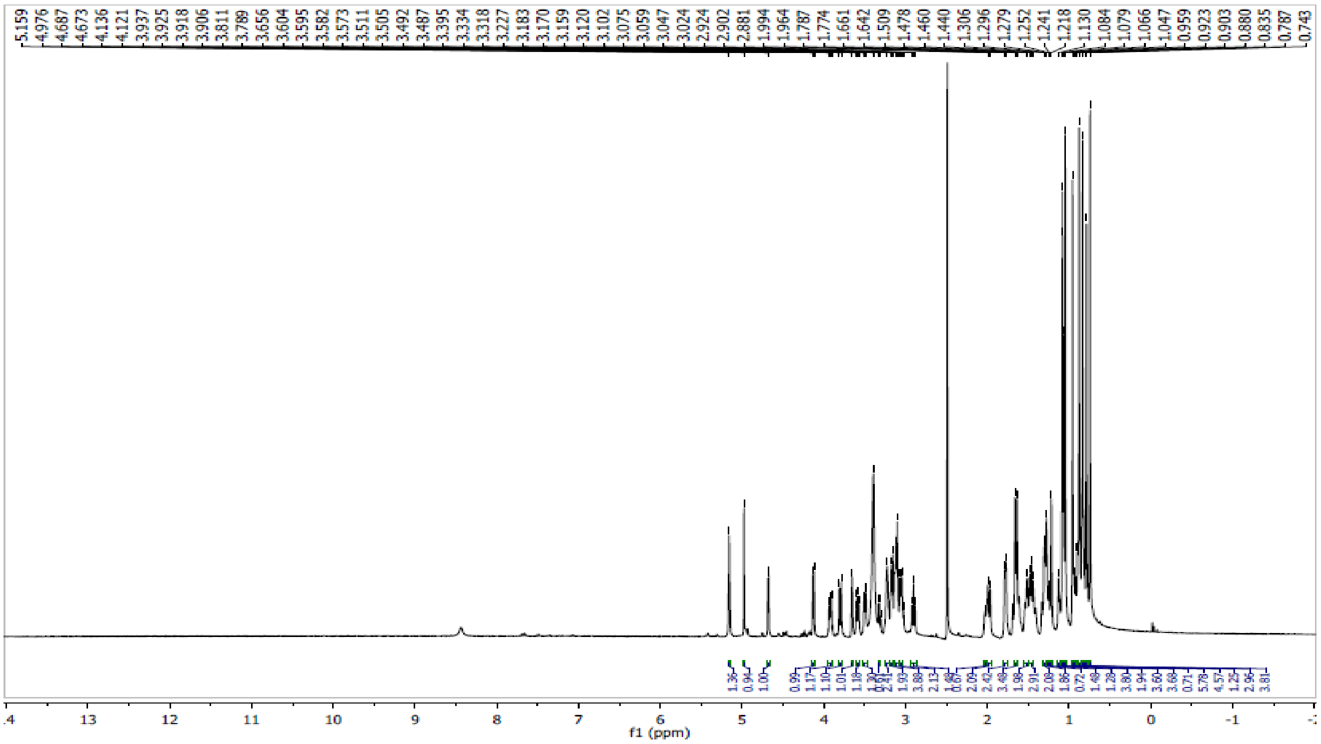

Figure S1. ^{1}H-NMR (500 MHz, DMSO-d_6) spectrum of compound **25**.

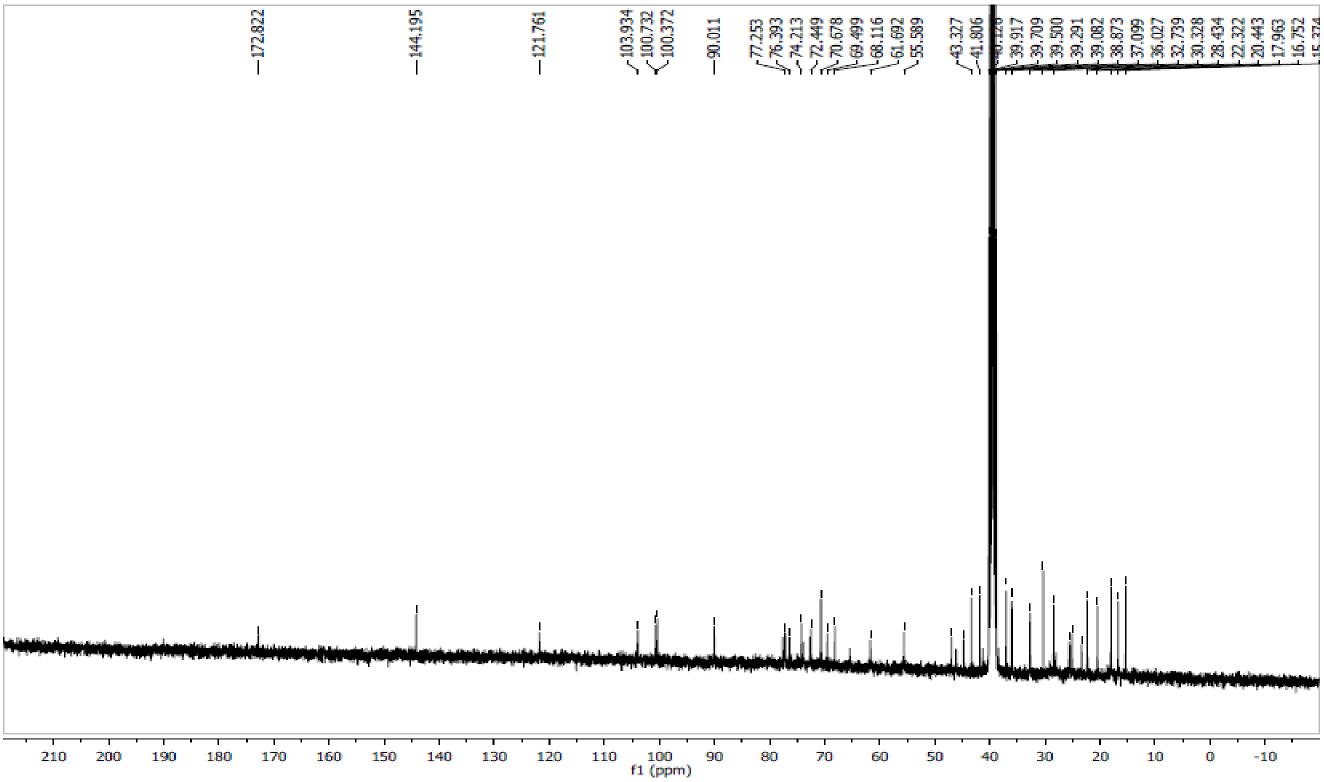

Figure S2. ^{13}C-NMR (100 MHz, DMSO-d_6) spectrum of compound **25**.

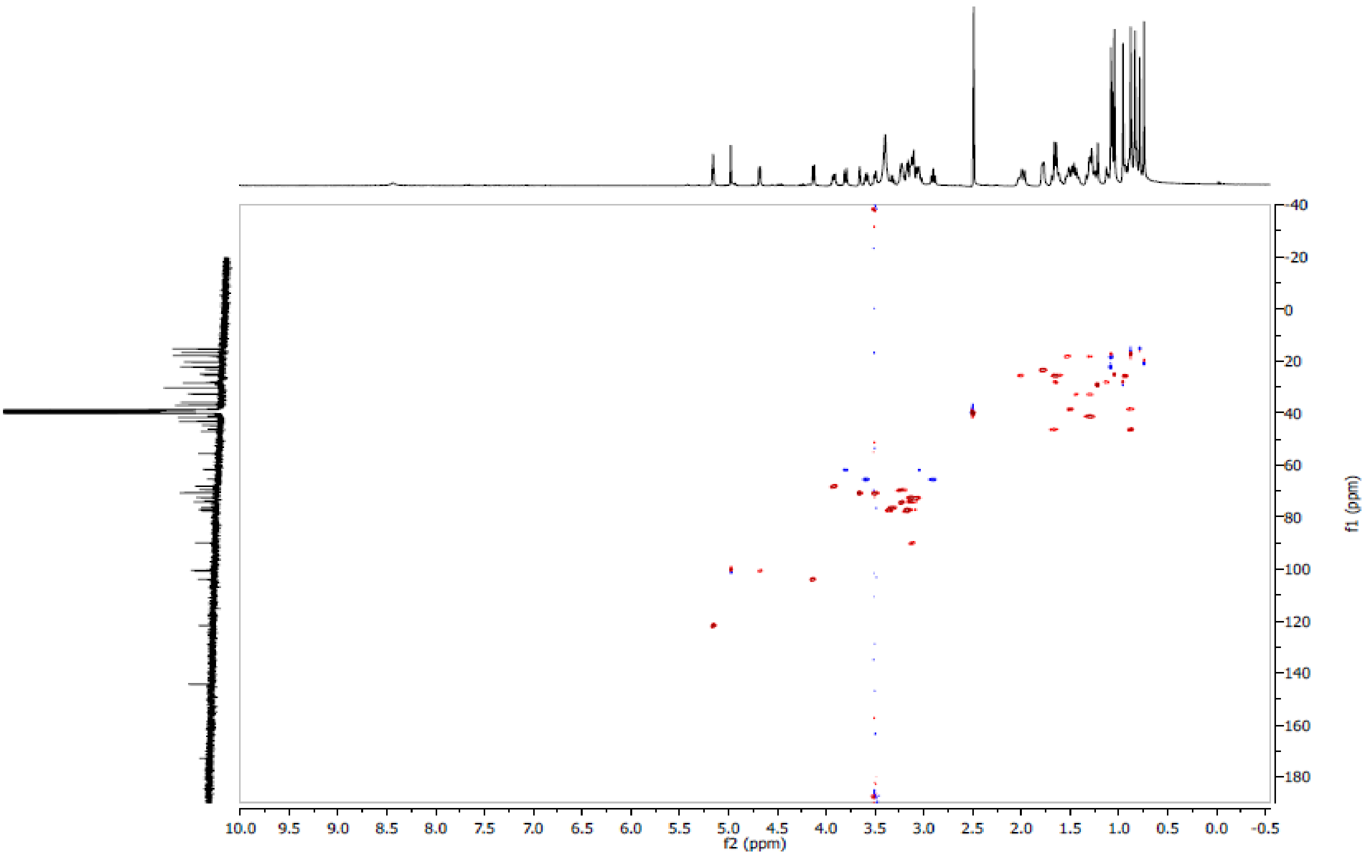

Figure S3. HSQC (500 x 125 MHz, DMSO-d_6) spectrum of compound **25.**

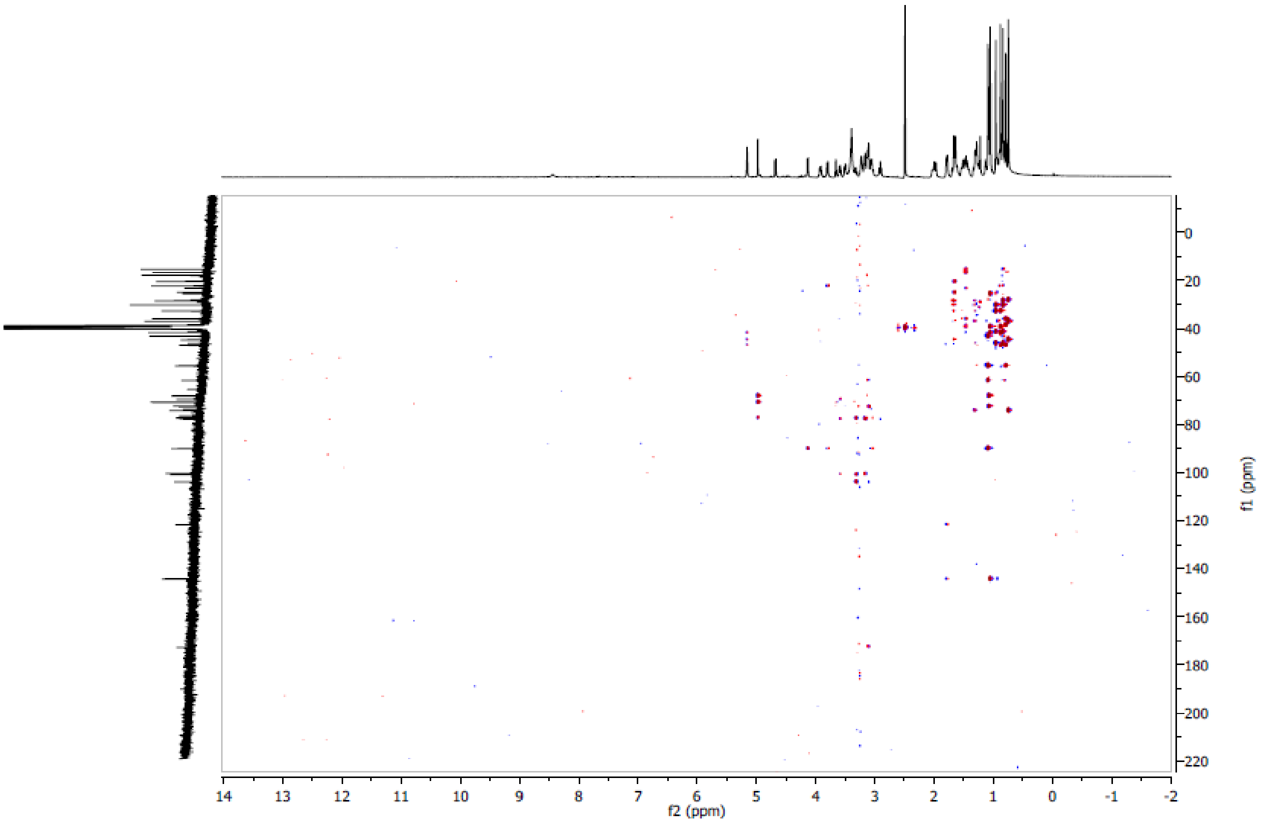

Figure S4. HMBC (500 x 125 MHz, DMSO-d_6) spectrum of compound **25**

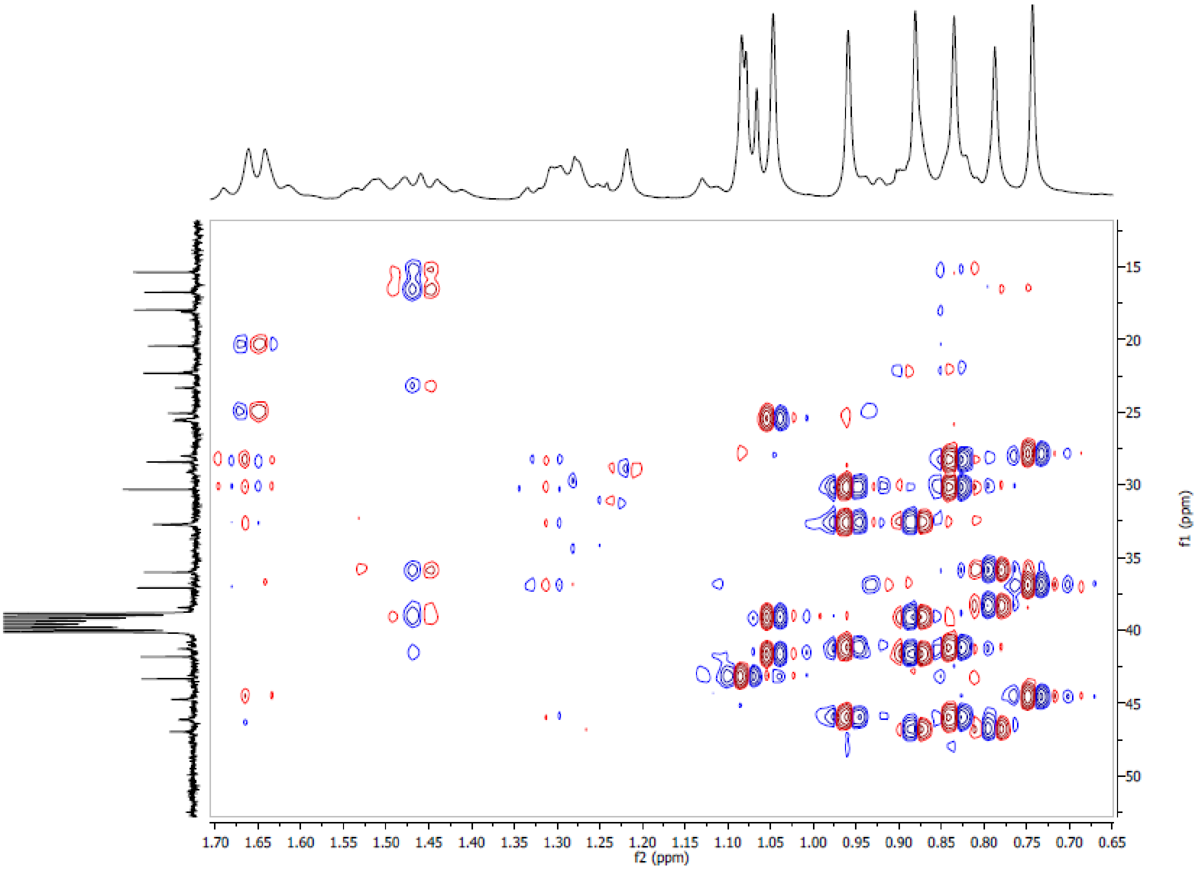

Figure S5. Expansion of HMBC (500 x 125 MHz, DMSO-d_6) spectrum of compound **25**.

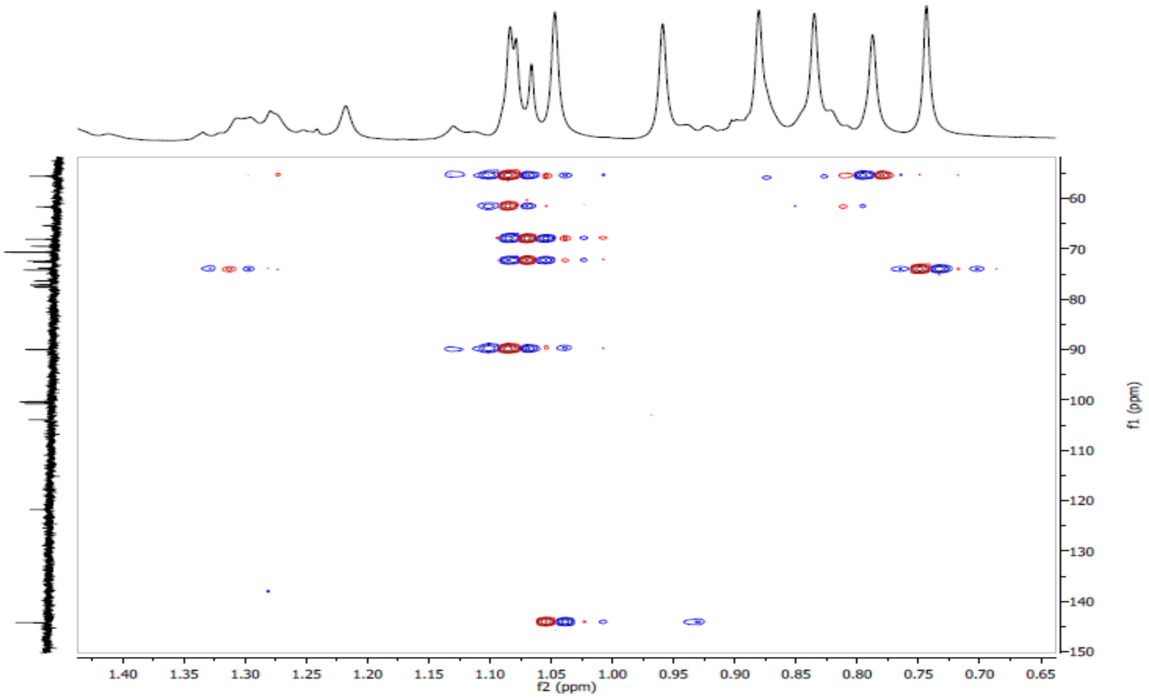

Figure S6. Expansion of HMBC (500 x 125 MHz, DMSO-d_6) spectrum of compound **25.**

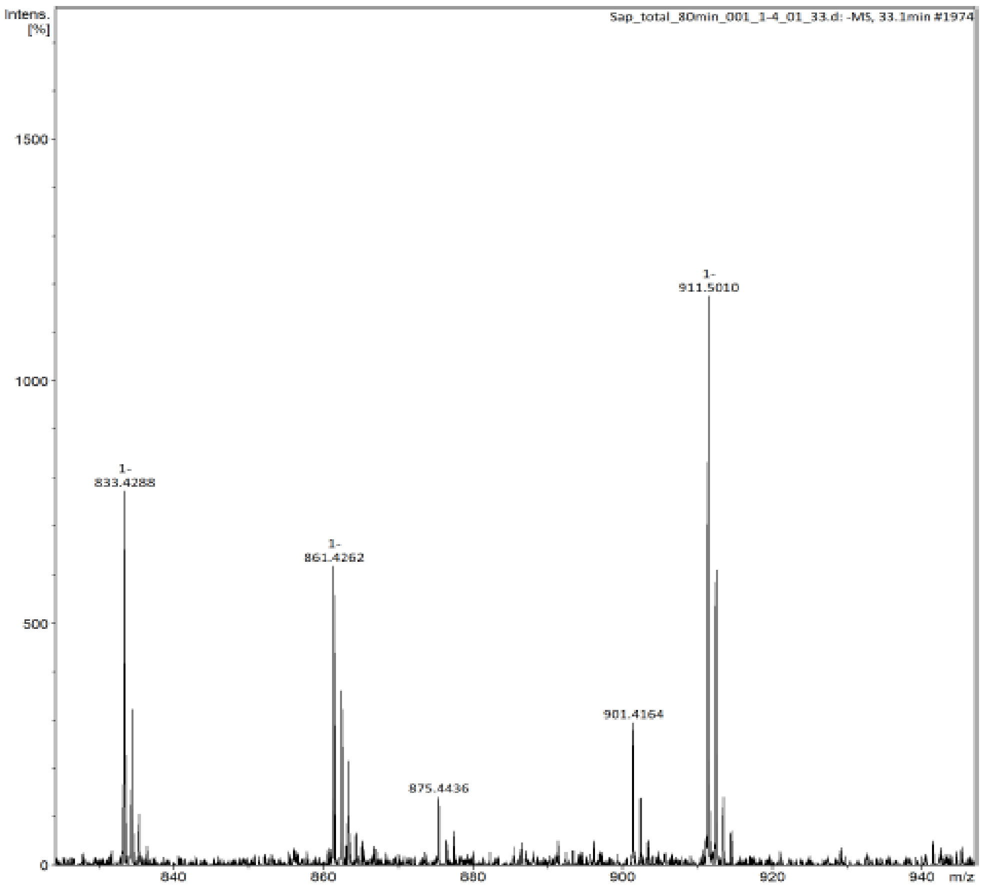

Figure S7. The HRESI-TOF-MS spectrum of compound **25.**

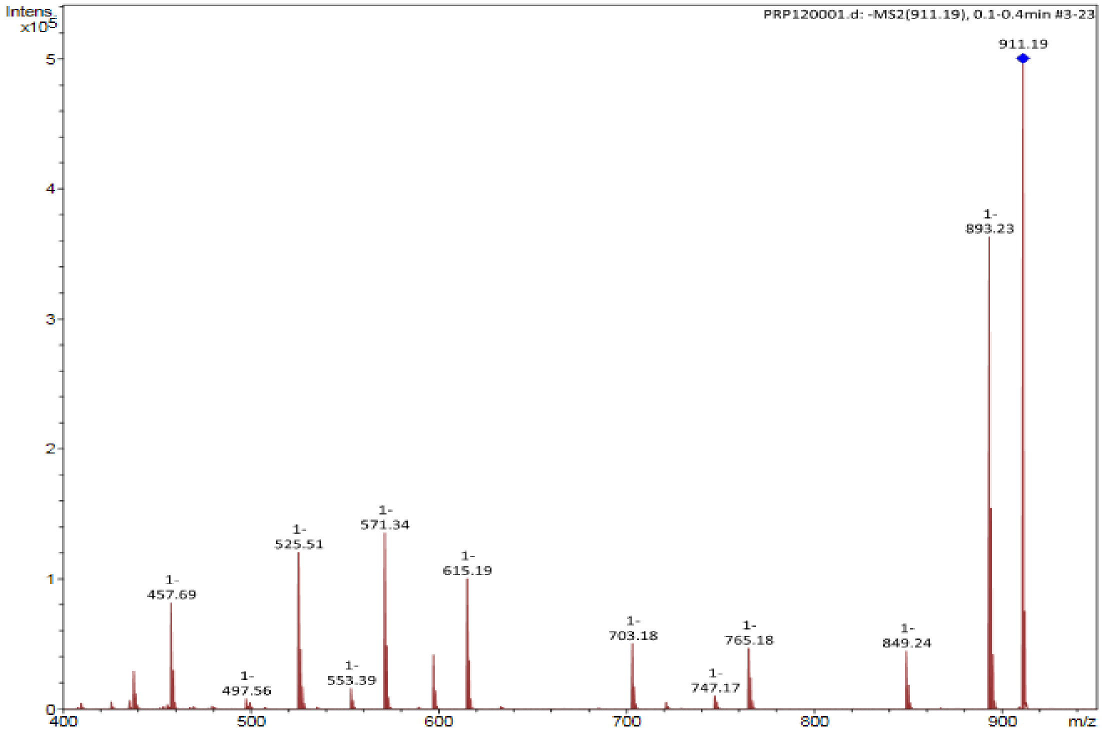

Figure S8. The ESI-MS/MS spectrum of compound **25.**

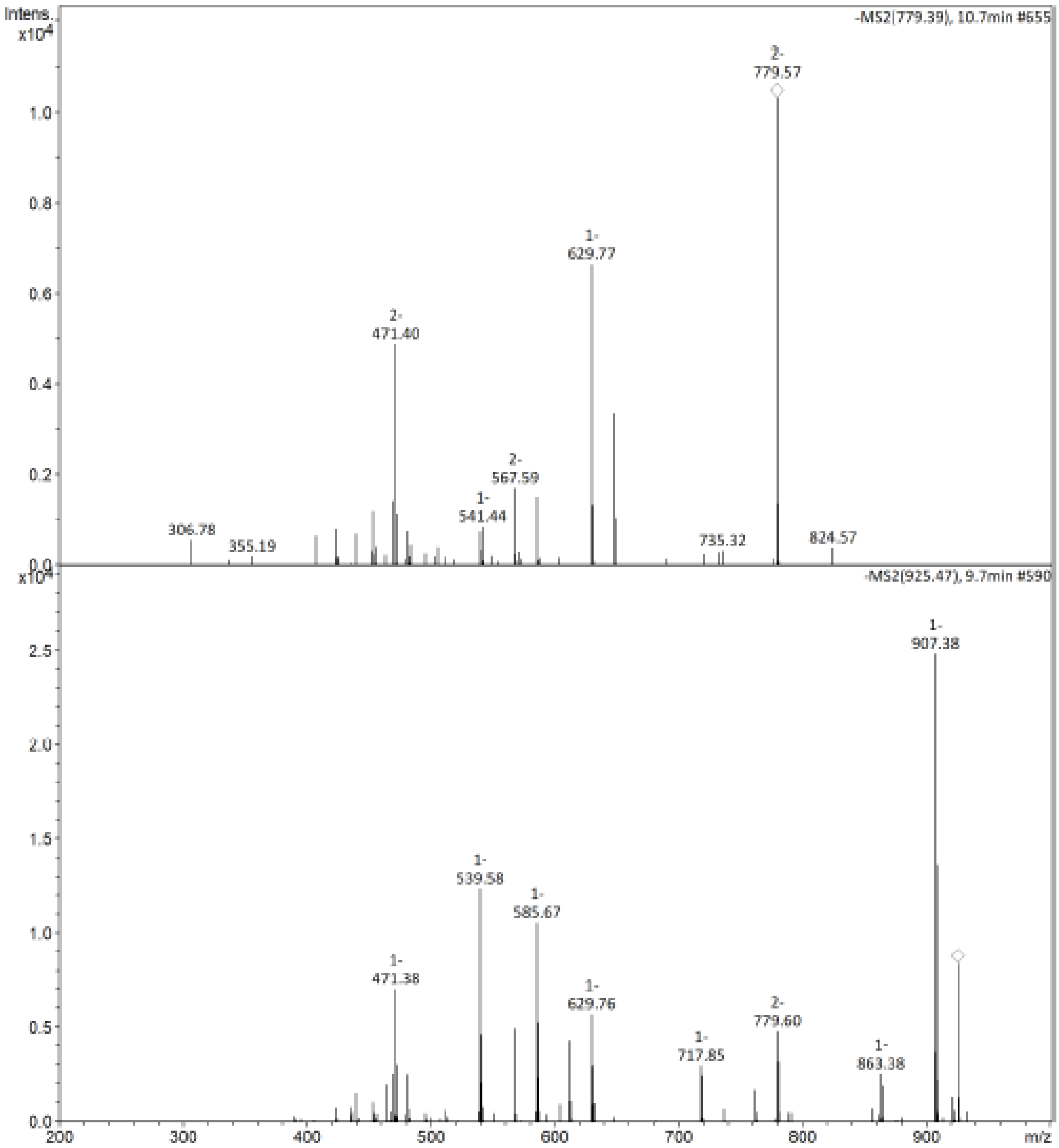

Figure S9. The ESI-MS/MS spectrum of compounds **1** and **2.**

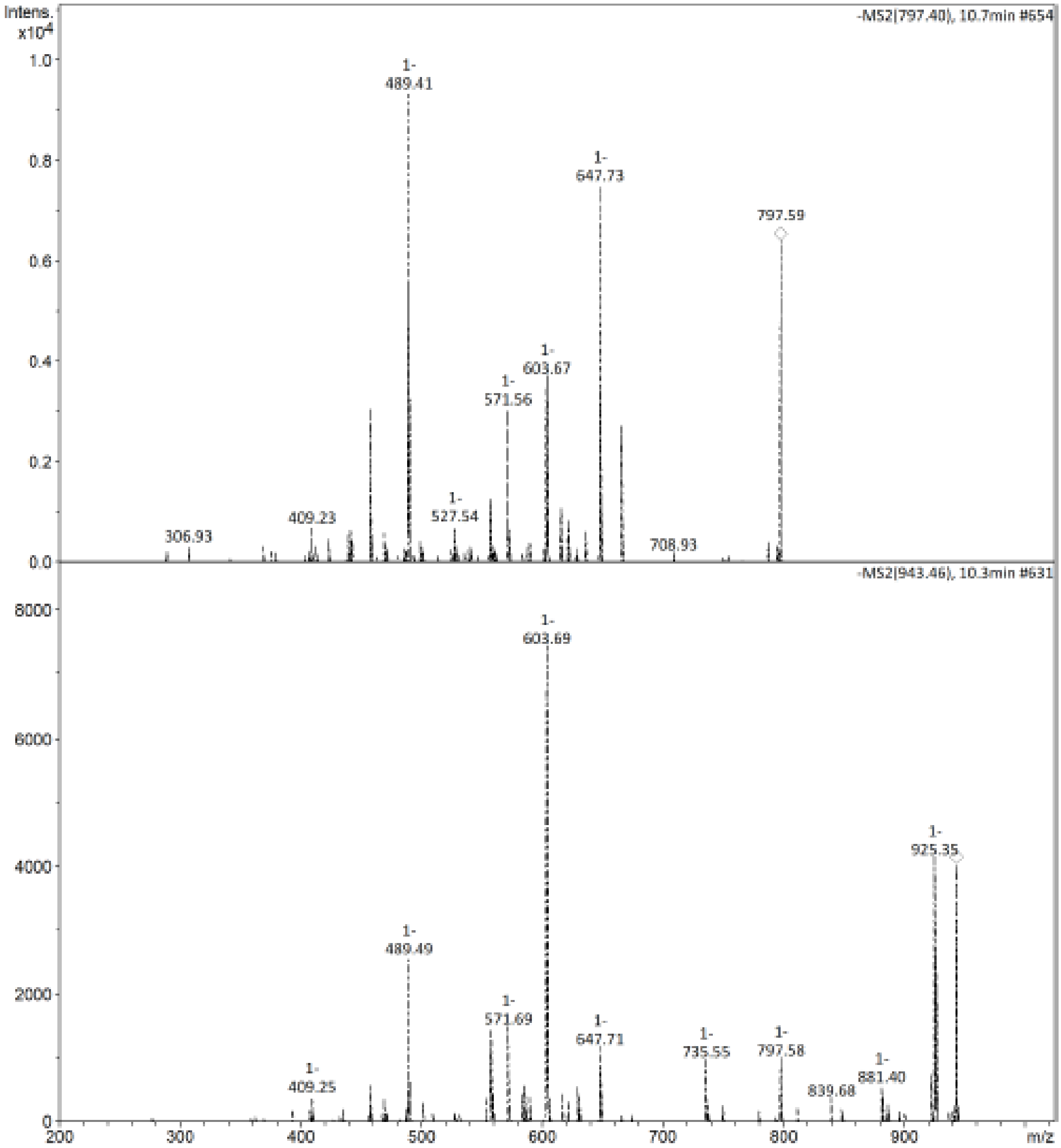

Figure S10. The ESI-MS/MS spectrum of compounds **3** and **4.**

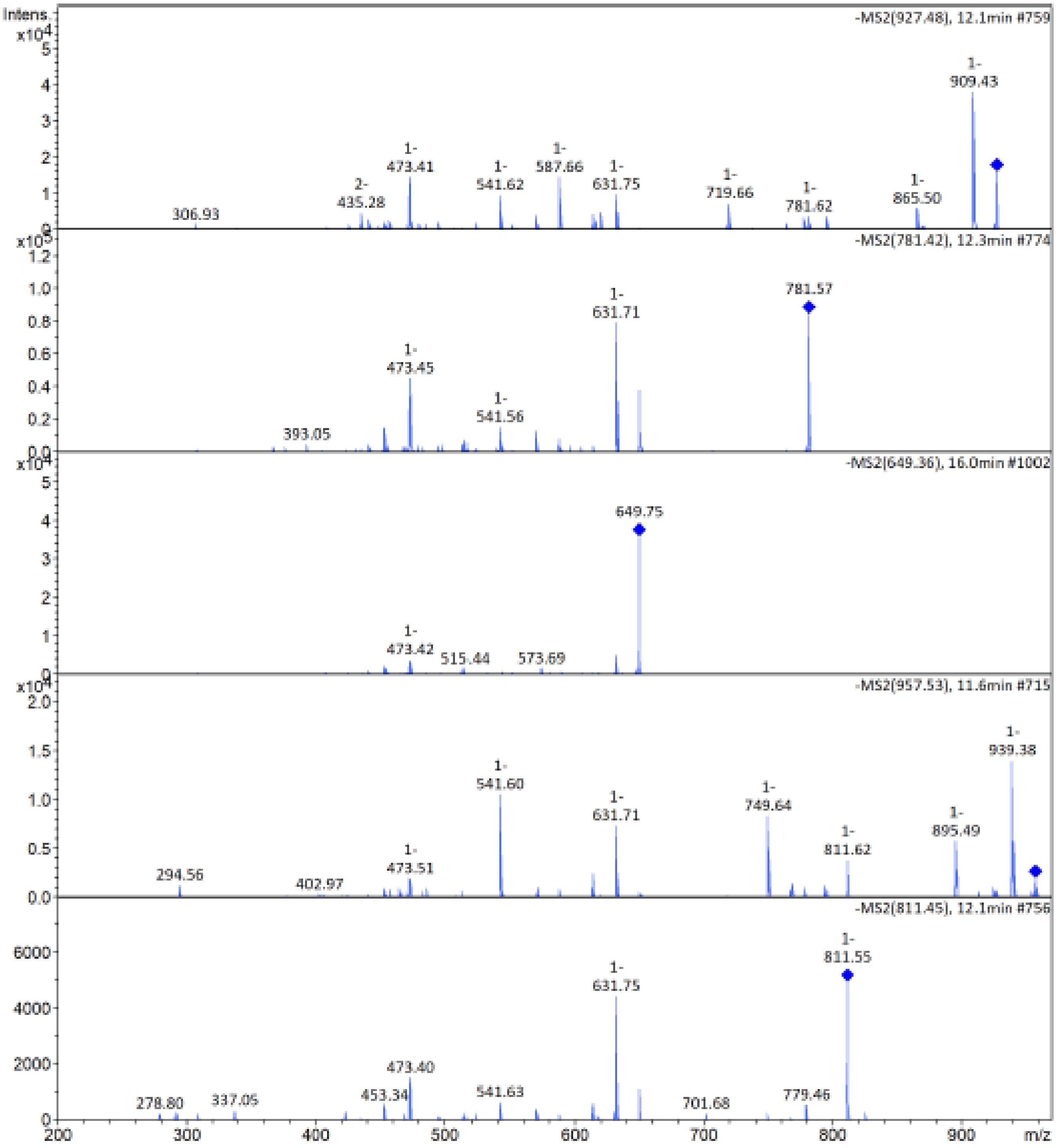

Figure S11. The ESI-MS/MS spectrum of compounds **5**, **6**, **7**, **8** and **14.**

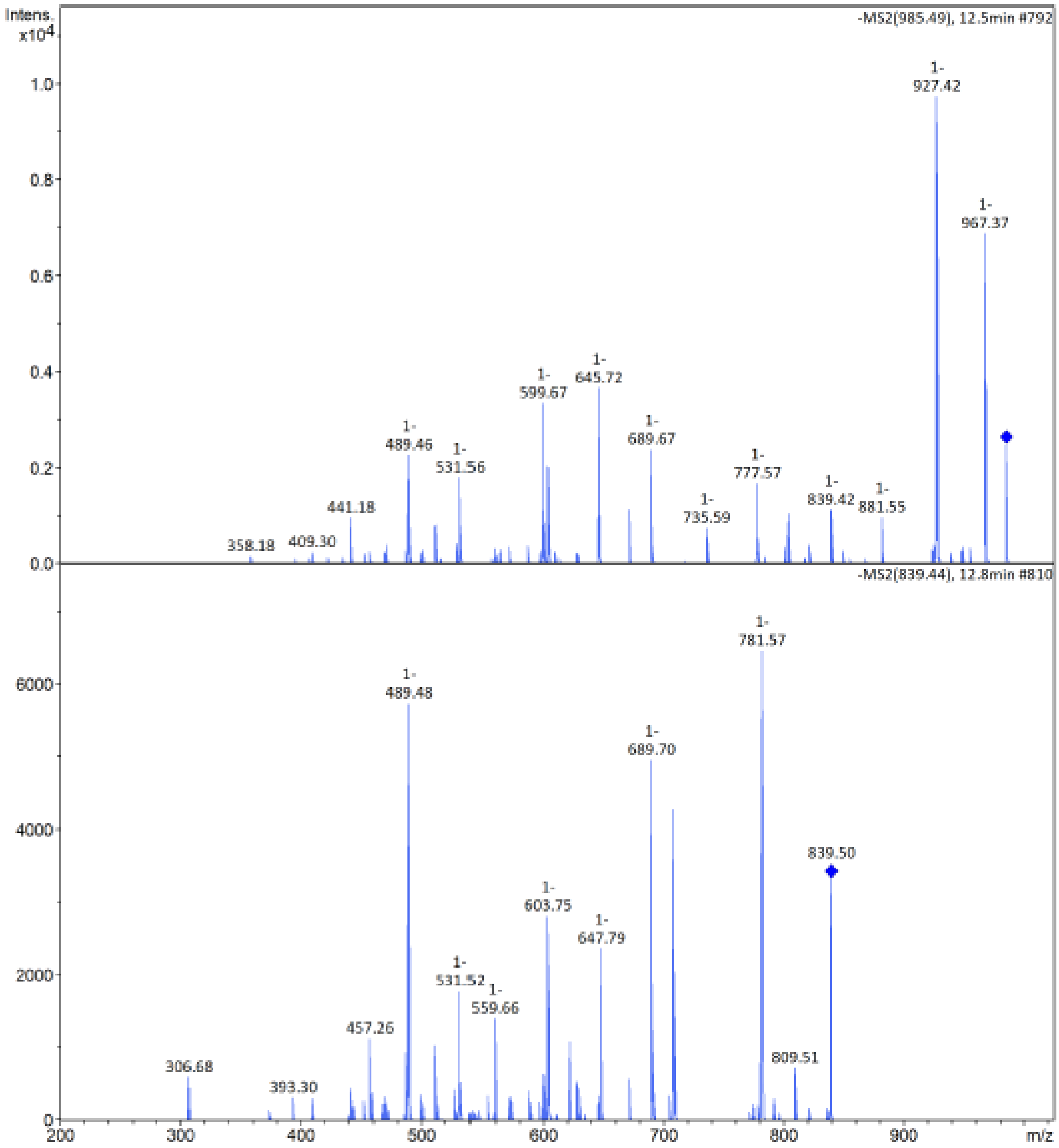

Figure S12. The ESI-MS/MS spectrum of compounds **9** and **10.**

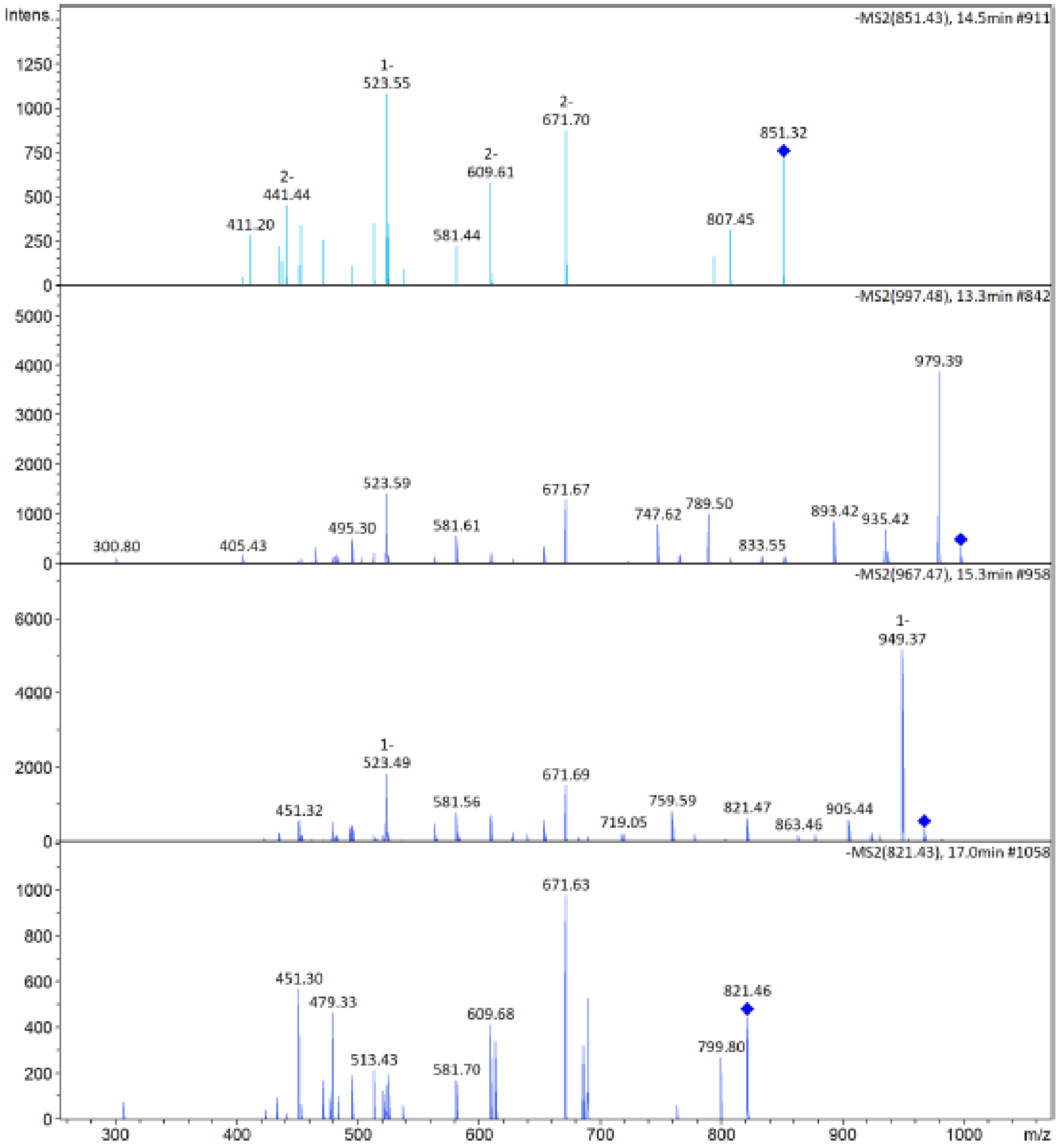

Figure S13. The ESI-MS/MS spectrum of compounds **11**, **12**, **13** and **15.**

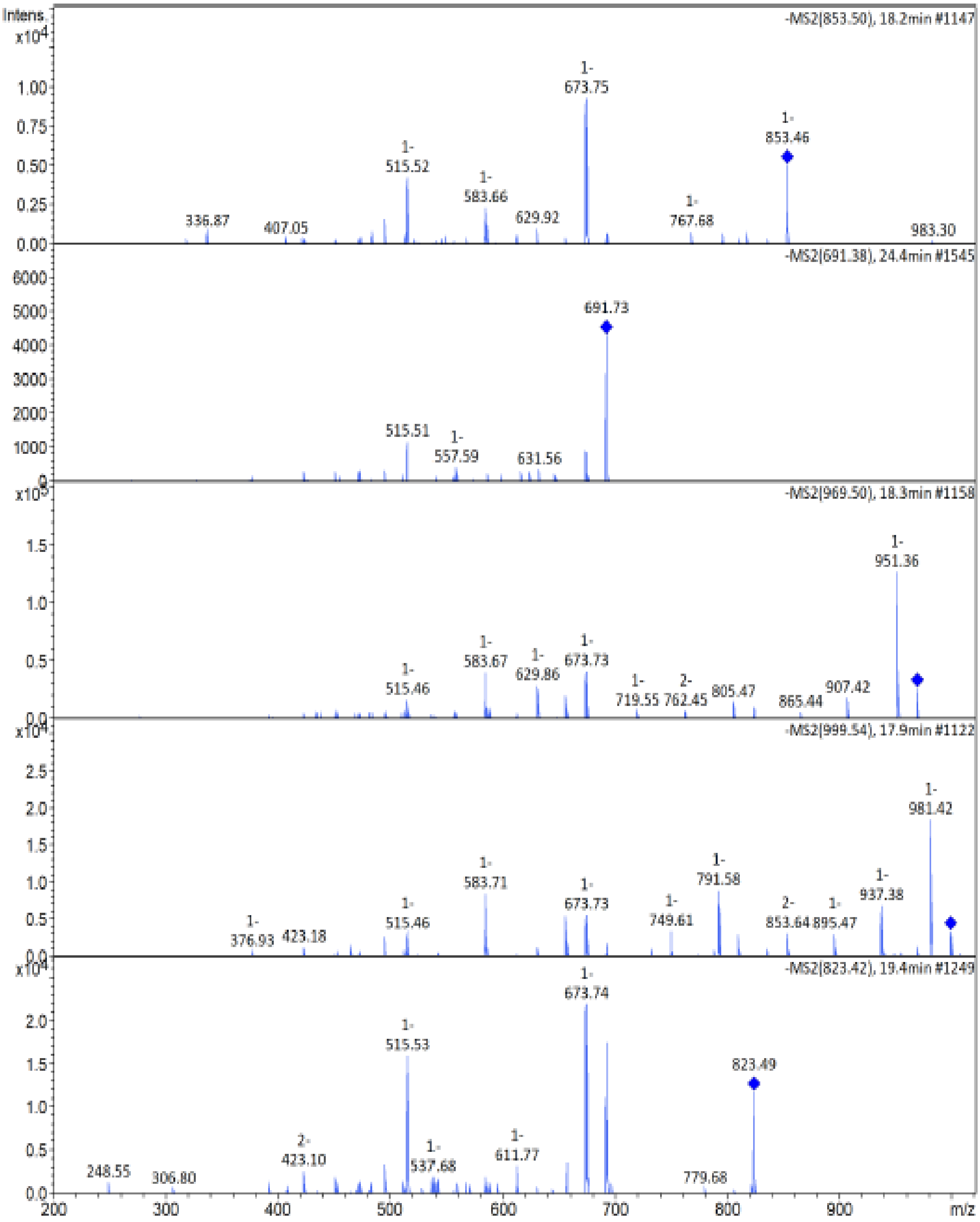

Figure S14. The ESI-MS/MS spectrum of compounds **16**, **17**, **18**, **19** and **22.**

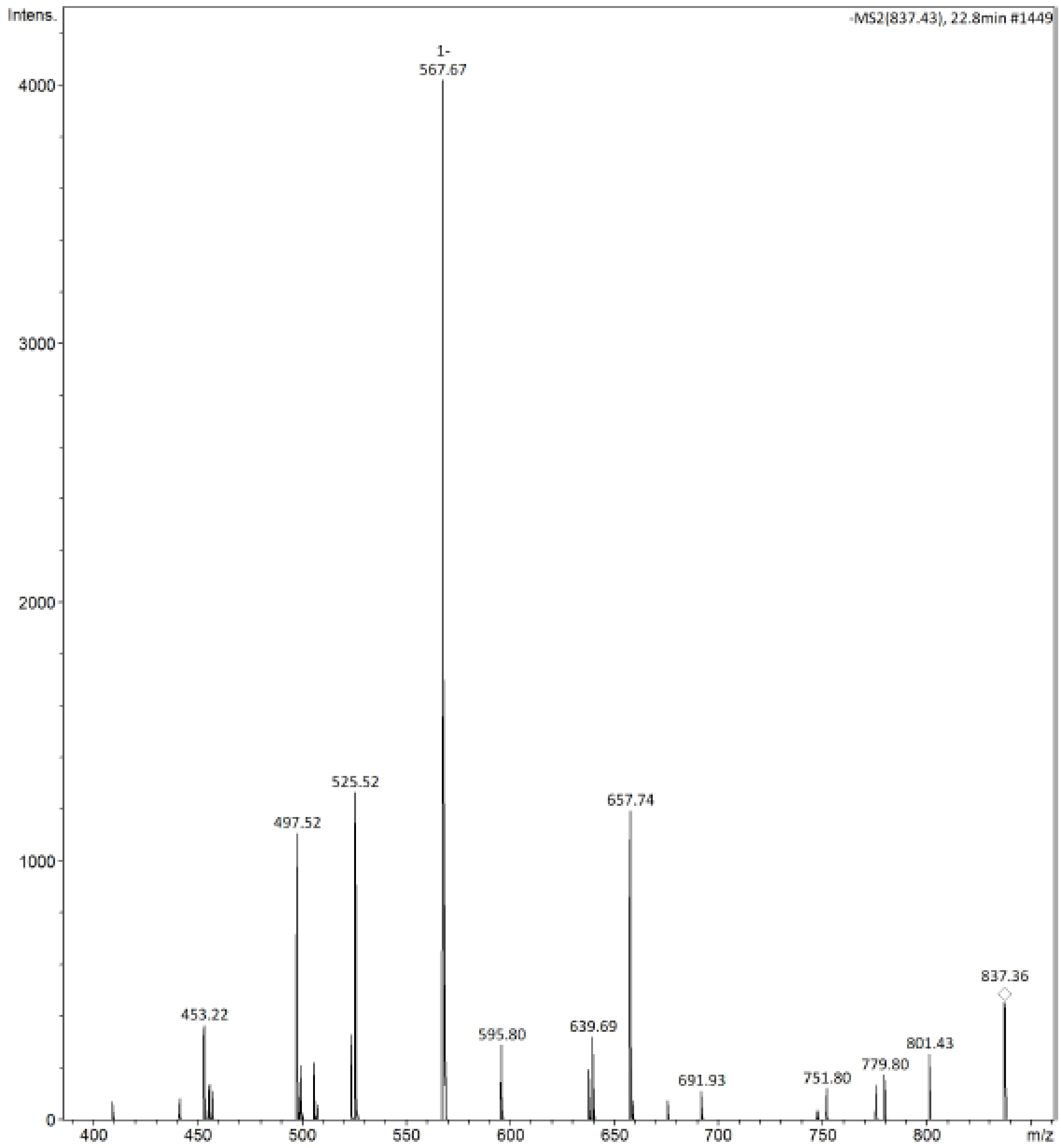

Figure S15. The ESI-MS/MS spectrum of compound **20.**

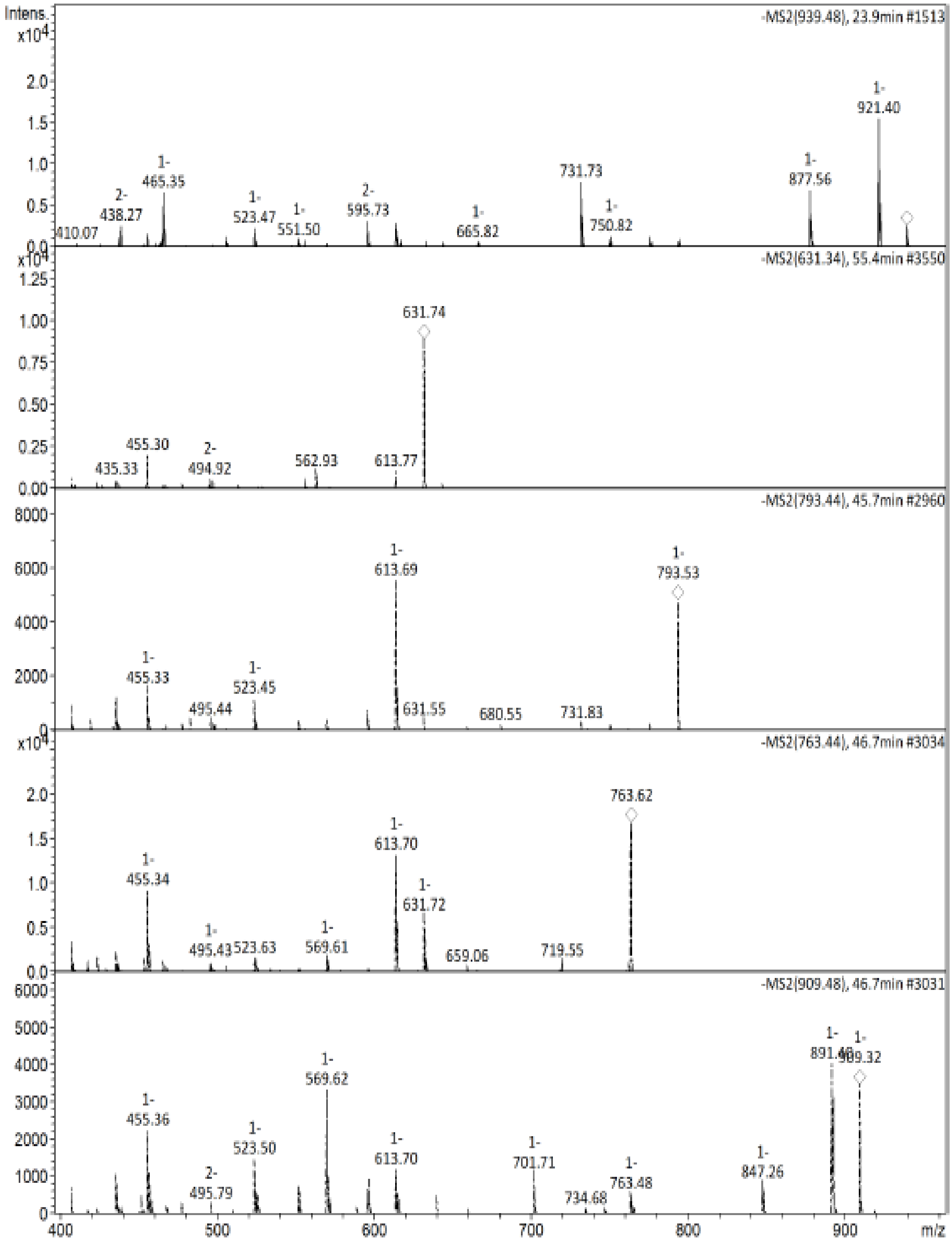

Figure S16. The ESI-MS/MS spectrum of compounds **21**, **31**, **32**, **33** and **35.**

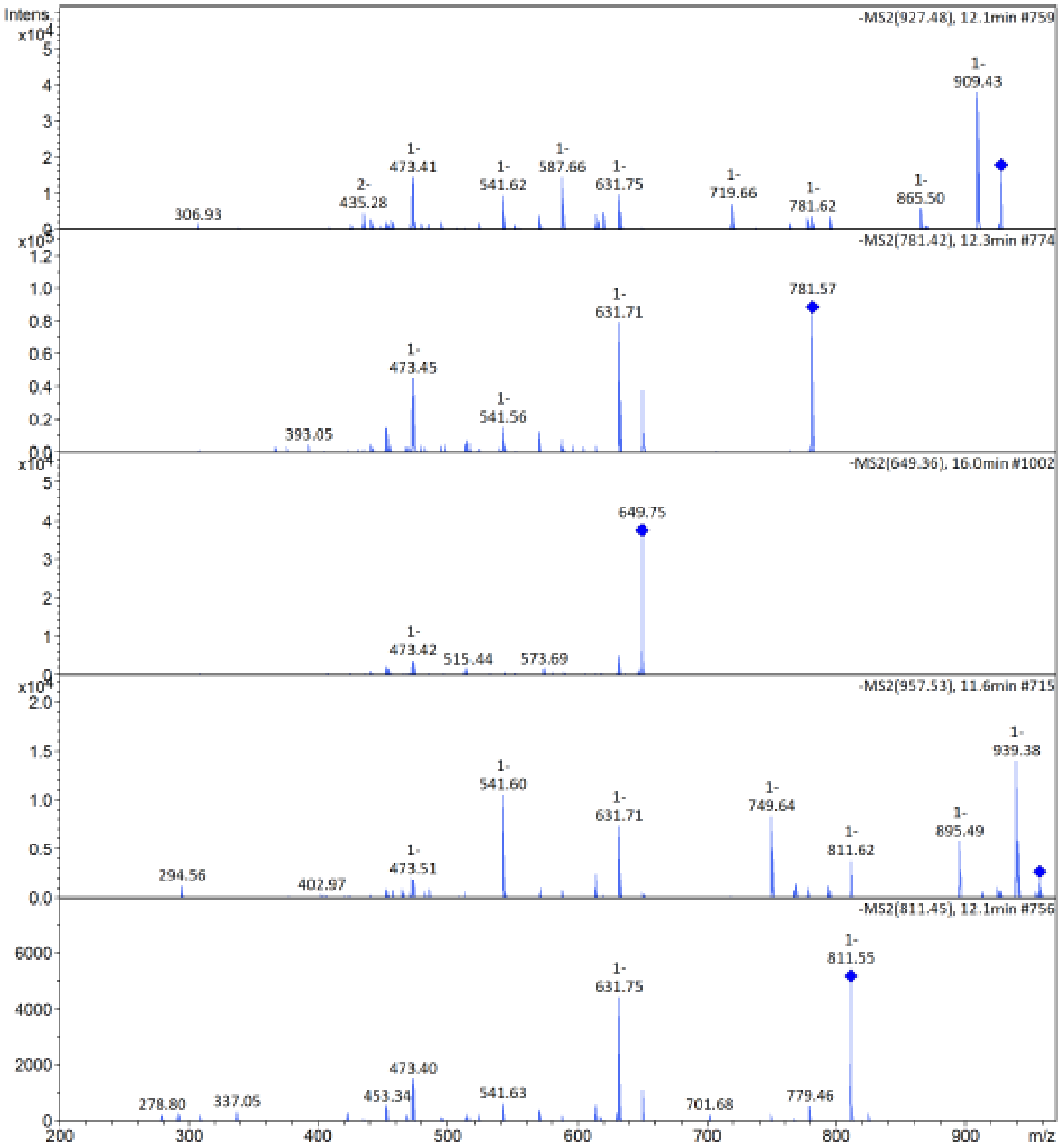

Figure S17. The ESI-MS/MS spectrum of compounds **22**, **24**, **25**, **26**, and **29.**

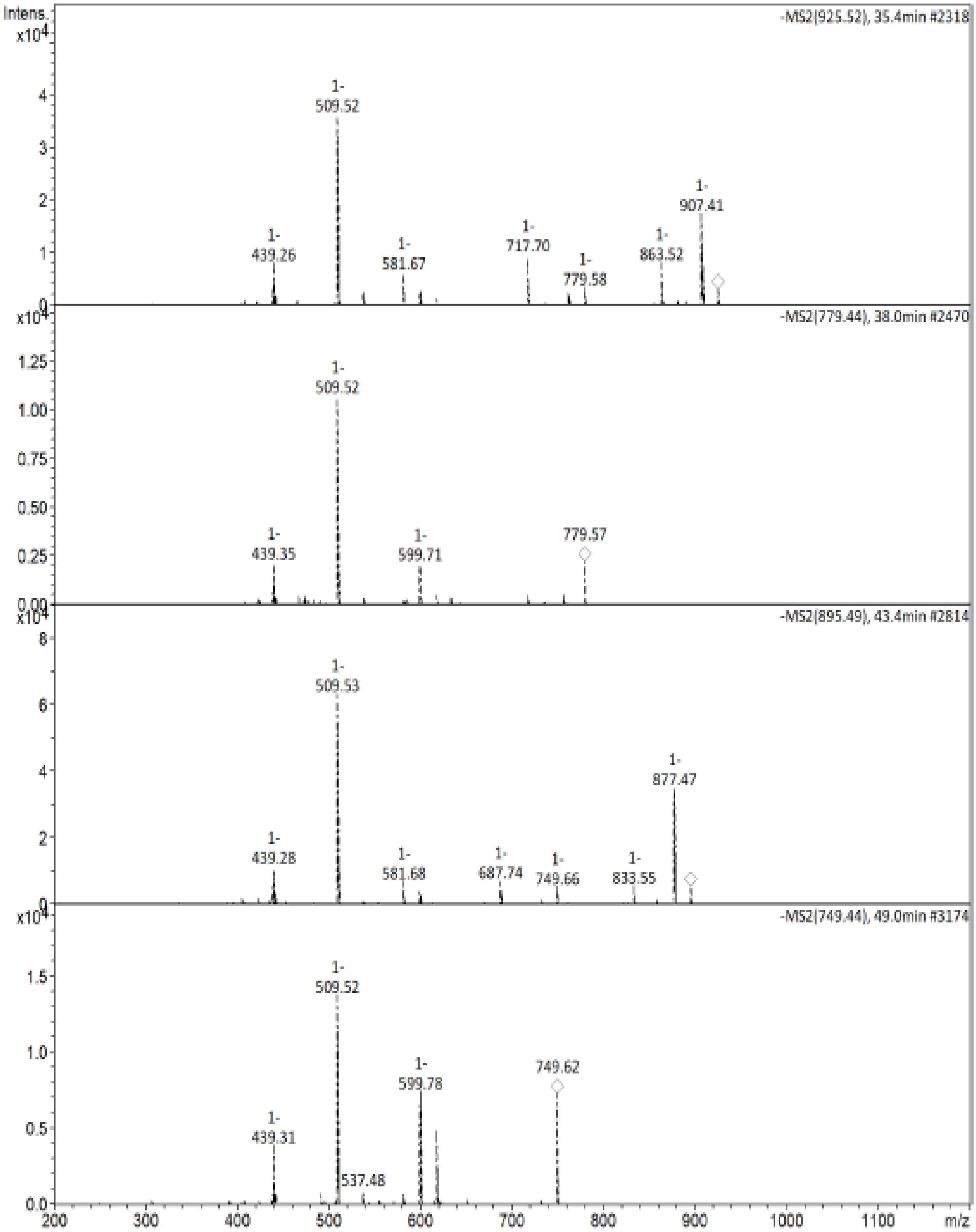

Figure S18. The ESI-MS/MS spectrum of compounds **27**, **28**, **30** and **34.**

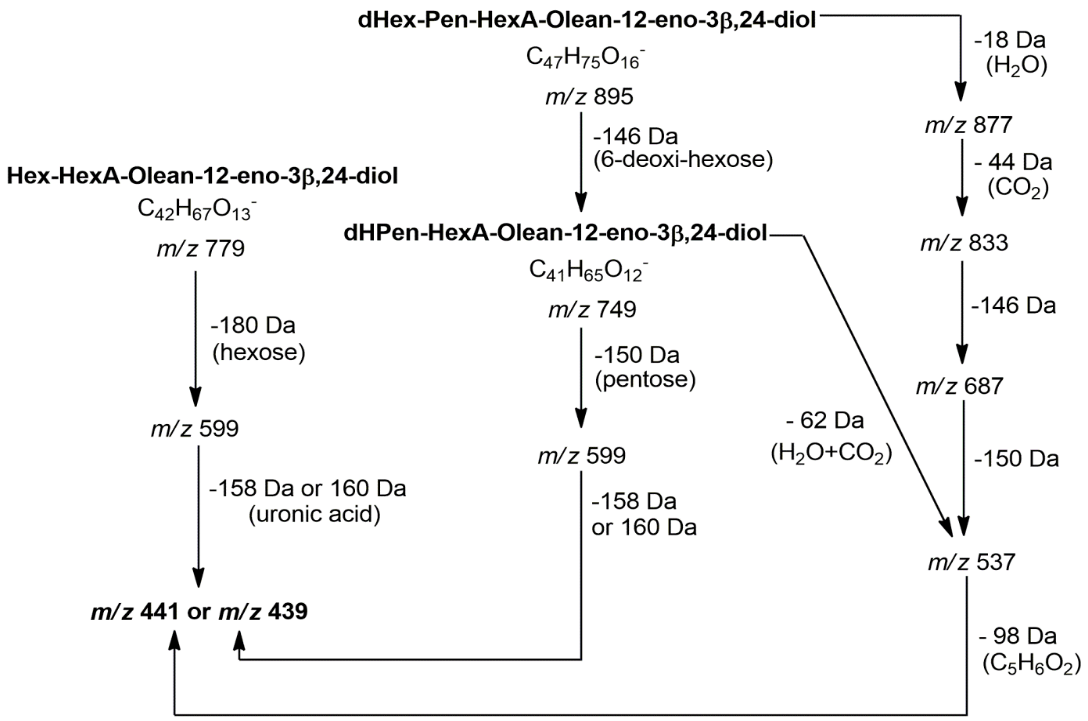

Figure S19. Fragmentation pathway of compounds **28** (*m/z* 779.4562), **30** (*m/z* 895.5043) and **34** (*m/z* 749.4485) having olean-12-ene-3β,diol (*m/z* 441 or 439) as aglycone in negative mode ESI-MS/MS.

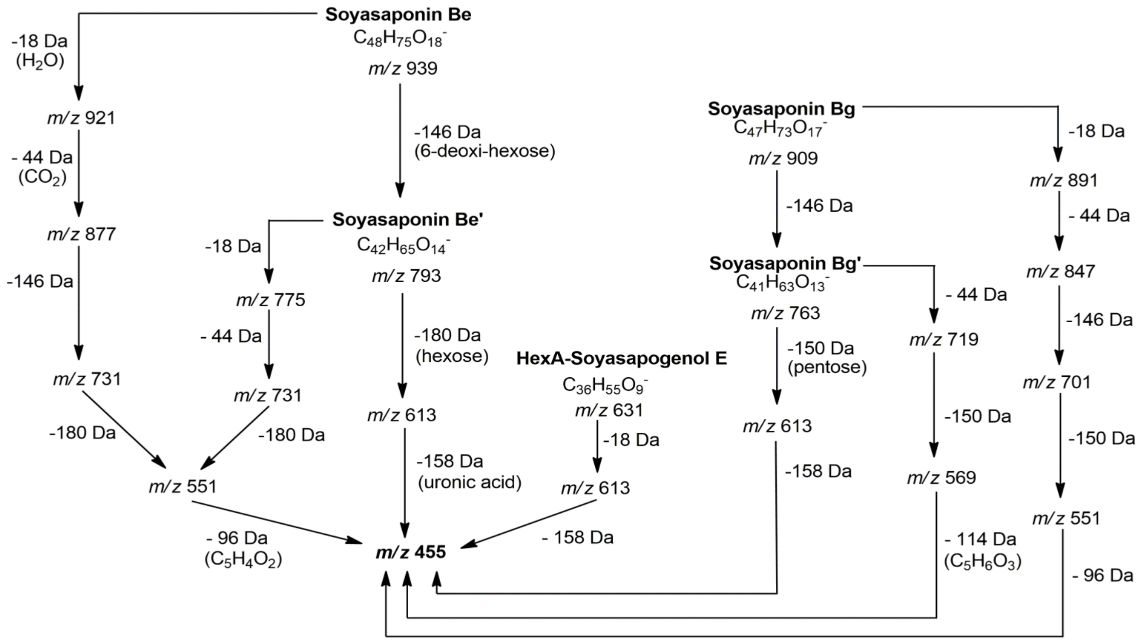

Figure S20. Fragmentation pathway of compounds **21** (*m/z* 939.4935), **31** (*m/z* 793.4348), **32** (*m/z* 909.4843), **33** (*m/z* 763.4286) and **35** (*m/z* 631.3856), which have soyasapogenol E (*m/z* 455) as aglycone, in negative mode ESI-MS/MS.

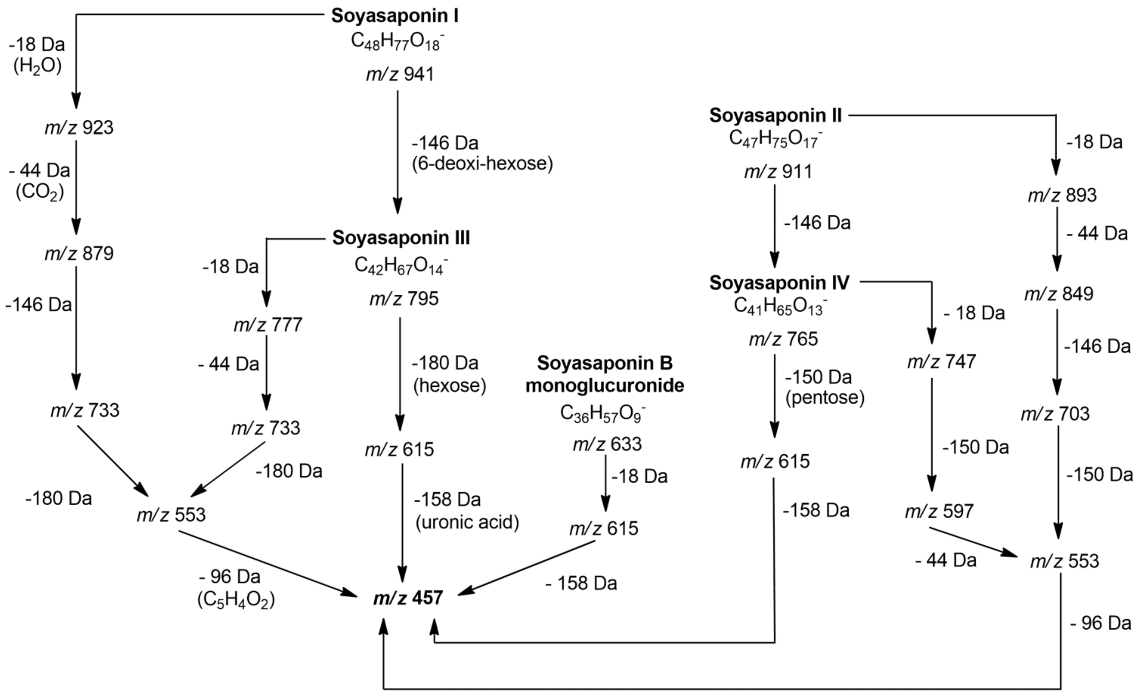

Figure S21. Fragmentation pathway of compounds **23** (*m/z* 941.5123), **24** (*m/z* 795.4520), **25** (*m/z* 911.5006), **26** (*m/z* 765.4418) and **29** (*m/z* 633.3998), which possess soyasapogenol B (*m/z* 457) as aglycone, in negative mode ESI-MS/MS.

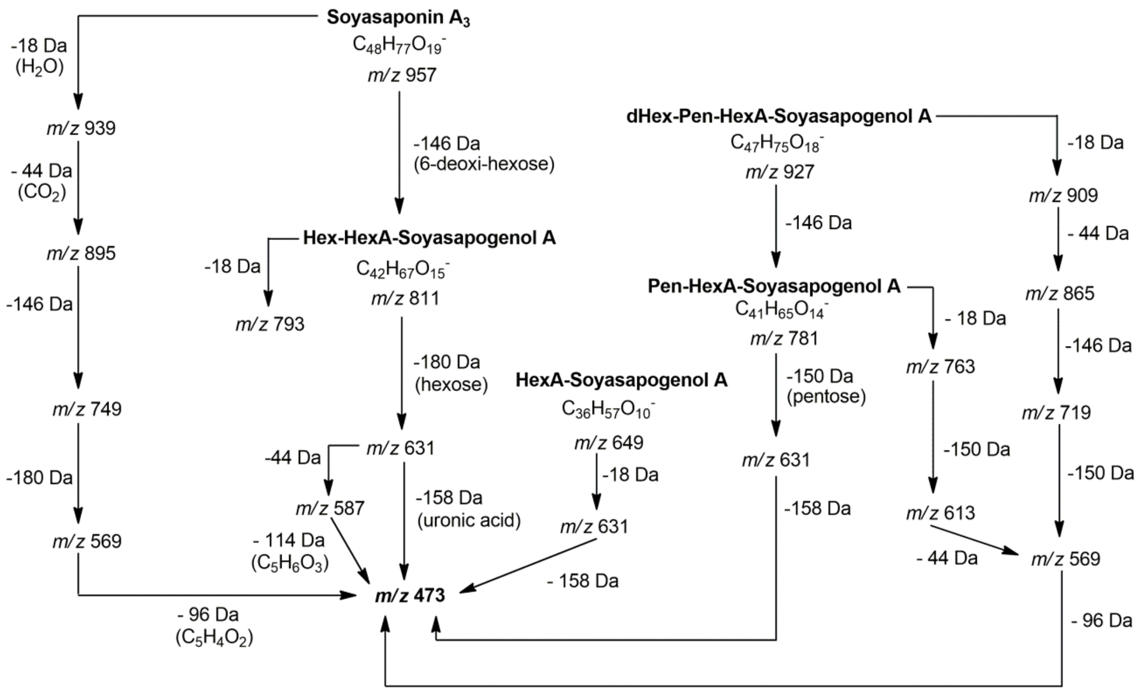

Figure S22. Fragmentation pathway of compounds **5** (*m/z* 957.5056), **8** (*m/z* 811.4459), **6** (*m/z* 927.4943), **7** (*m/z* 781.4350) and **14** (*m/z* 649.3933), which have soyasapogenol A (*m/z* 473) as aglycone, in negative mode ESI-MS/MS.

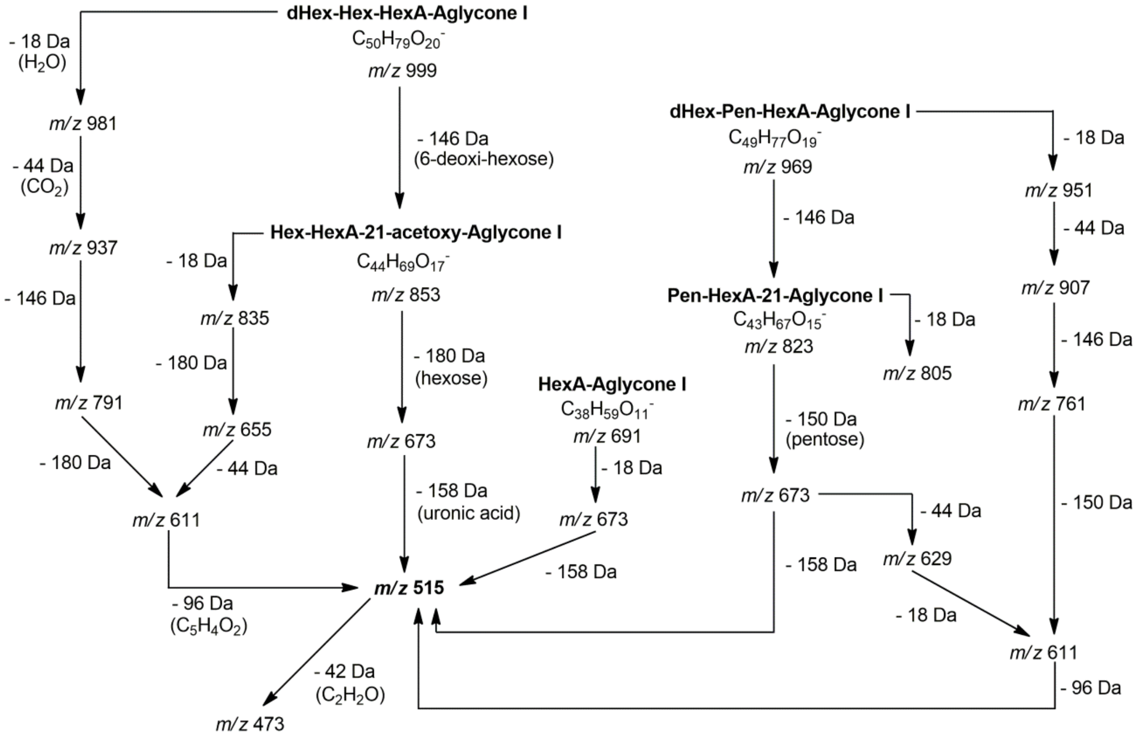

Figure S23 Fragmentation pathway of compounds **16** (*m/z* 999.5135), **18** (*m/z* 853.4559), **17** (*m/z* 969.5074), **19** (*m/z* 823.4463) and **22** (*m/z* 691.4042), which have Aglycone I (*m/z* 515) as aglycone, in negative mode ESI-MS/MS.

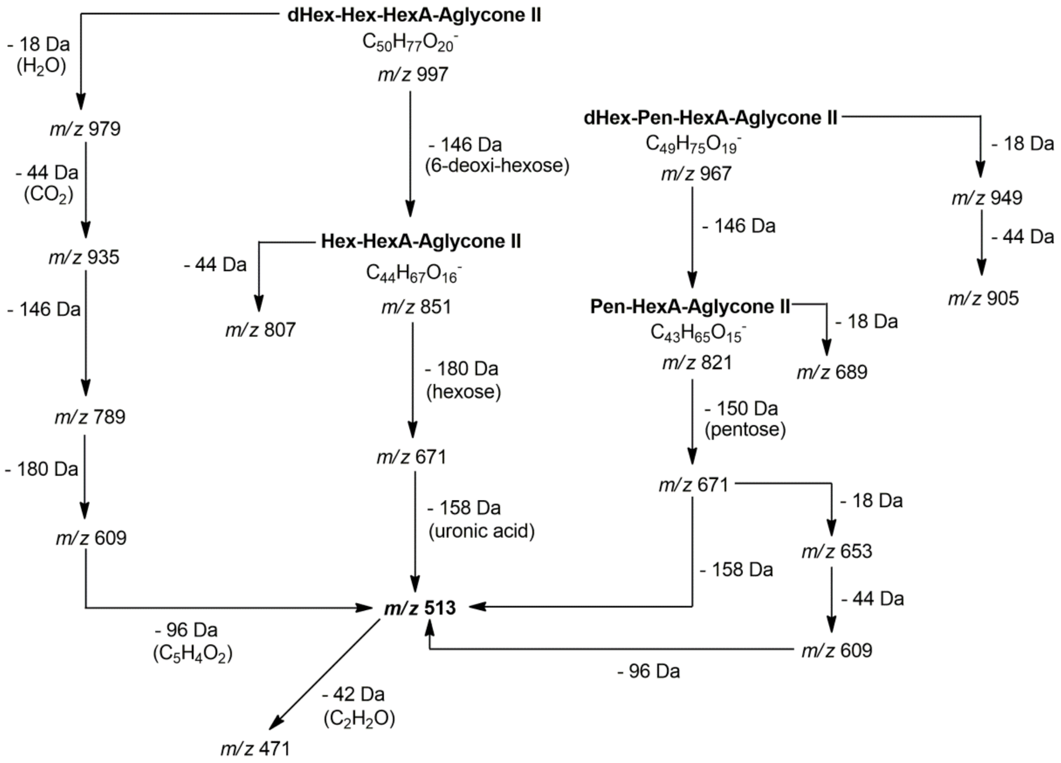

Figure S24. Fragmentation pathway of compounds **11** (*m/z* 997.5022), **12** (*m/z* 851.4412), **13** (*m/z* 967.4901) and **15** (*m/z* 821.4303), which have Aglycone II (*m/z* 513) as aglycone, in negative mode ESI-MS/MS.

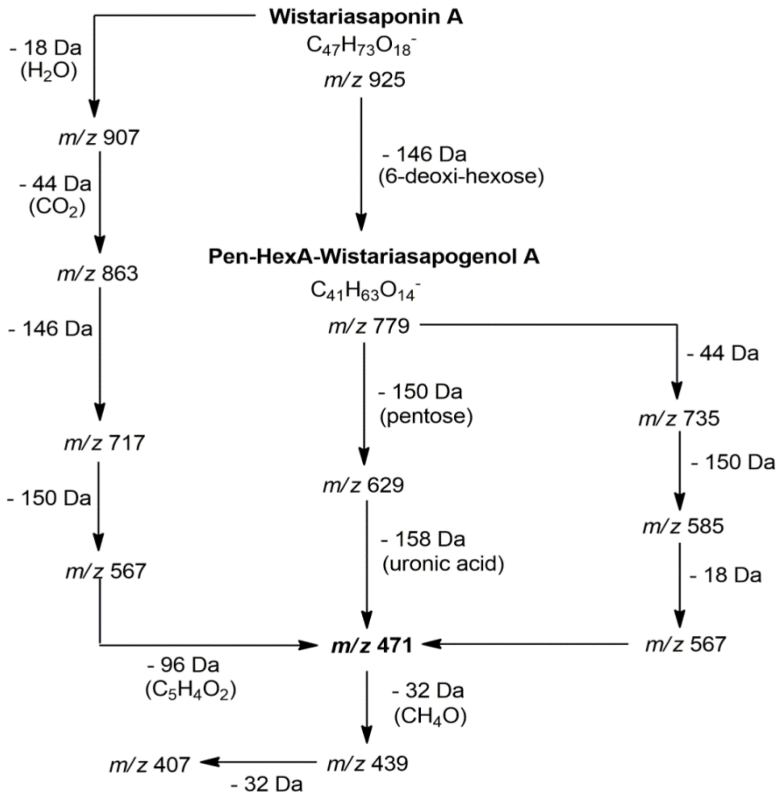

Figure S25. Fragmentation pathway of compounds **1** (*m/z* 925.4800) and **2** (*m/z* 779.4239), which have wistariasapogenol A (*m/z* 471) as aglycone, in negative mode ESI-MS/MS.

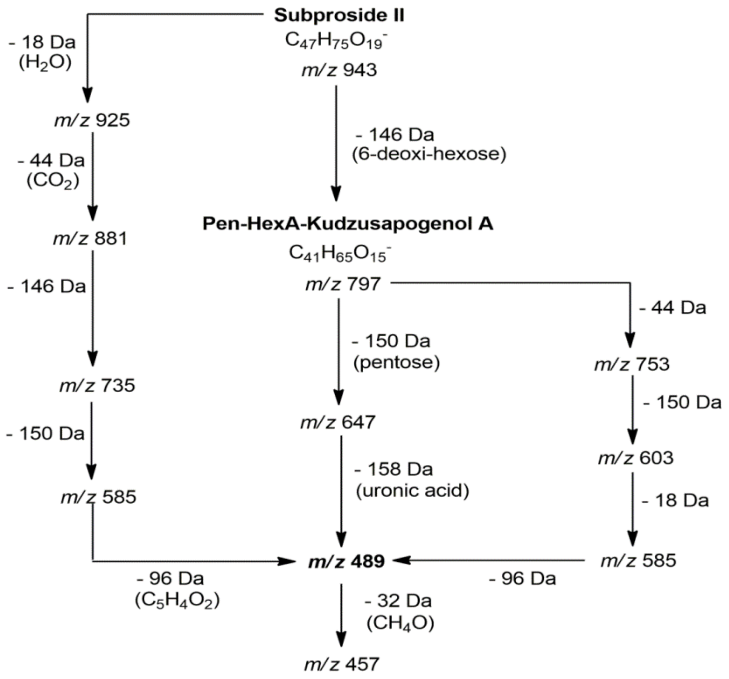

Figure S26. Fragmentation pathway of compounds **3** (*m/z* 797.4310) and **4** (*m/z* 943.4912), which have kudzusapogenol A (*m/z* 485) as aglycone, in negative mode ESI-MS/MS.

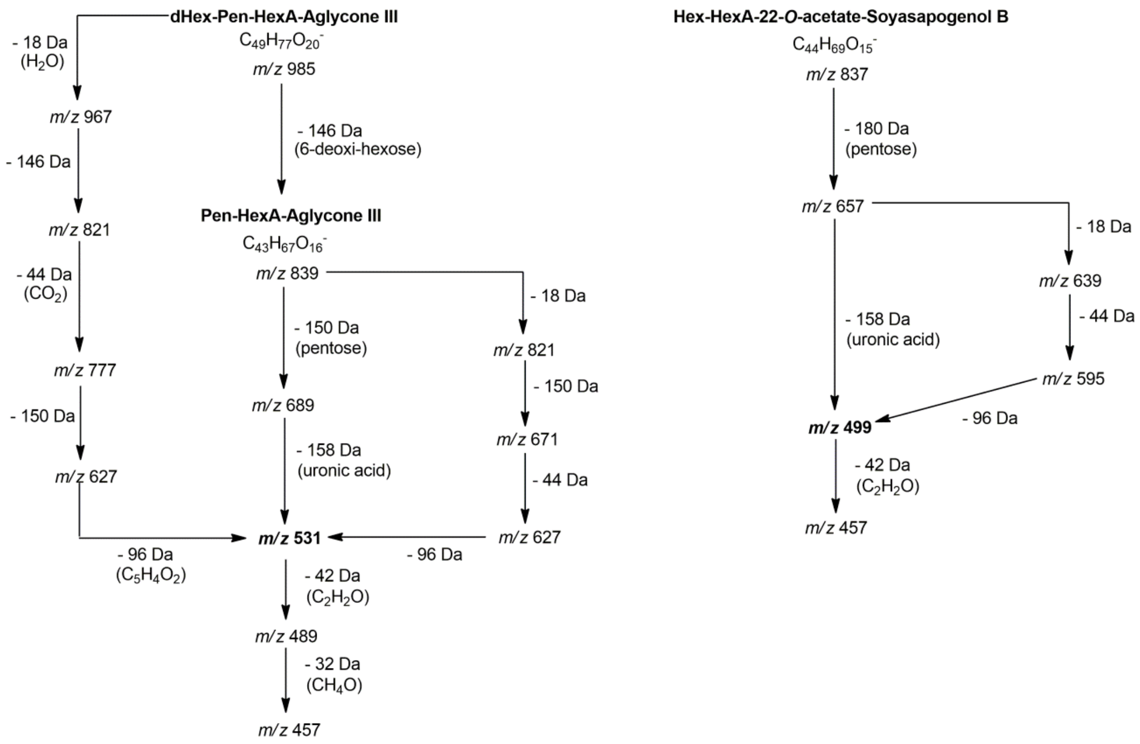

Figure S27. Fragmentation pathway of compounds **9** (*m/z* 985.4961) and **10** (*m/z* 839.4404) having Aglycone III (*m/z* 531) and that of compound **20** (*m/z* 837.4603), which has 22-*O*-acetate-soyasapogenol B (*m/z* 499) as aglycone, in negative mode ESI-MS/MS.
